# Development of a COX-2-Selective Fluorescent Probe for the Observation of Early Intervertebral Disc Degeneration

**DOI:** 10.3390/jfb14040192

**Published:** 2023-03-29

**Authors:** Cheol Ho Heo, Eun Ji Roh, Jaehee Kim, Hyemin Choi, Ho Yeon Jang, Giseong Lee, Chang Su Lim, Inbo Han

**Affiliations:** 1 Department of Applied Chemistry, Kookmin University, Seoul 02707, Republic of Korea; 2 Pure Chem Co., Ltd., Knu Start-up CUBE, Chunchenon 24341, Republic of Korea; 3 Department of Neurosurgery, CHA University School of Medicine, CHA Bundang Medical Center, Seongnam-si 13496, Republic of Korea; 4 Department of Biomedical Science, CHA University School of Medicine, CHA Bundang Medical Center, Seongnam-si 13496, Republic of Korea; 5 College of General Education, Kookmin University, Seoul 02707, Republic of Korea

**Keywords:** intervertebral disc, fluorescent probe, cyclooxygenase-2 (COX-2), diagnosis of intervertebral disc (IVD) degeneration, bioimaging, spinal disease

## Abstract

Cyclooxygenase-2 (COX-2) is a biomolecule known to be overexpressed in inflammation. Therefore, it has been considered a diagnostically useful marker in numerous studies. In this study, we attempted to assess the correlation between COX-2 expression and the severity of intervertebral disc (IVD) degeneration using a COX-2-targeting fluorescent molecular compound that had not been extensively studied. This compound, indomethacin-adopted benzothiazole-pyranocarbazole (**IBPC1**), was synthesized by introducing indomethacin—a compound with known selectivity for COX-2—into a phosphor with a benzothiazole-pyranocarbazole structure. **IBPC1** exhibited relatively high fluorescence intensity in cells pretreated with lipopolysaccharide, which induces inflammation. Furthermore, we observed significantly higher fluorescence in tissues with artificially damaged discs (modeling IVD degeneration) compared to normal disc tissues. These findings indicate that **IBPC1** can meaningfully contribute to the study of the mechanism of IVD degeneration in living cells and tissues and to the development of therapeutic agents.

## 1. Introduction

Intervertebral disc (IVD) degeneration is a medical condition that causes chronic pain due to disc damage [1,2]. This low back pain contributes large economic and medical burdens to modern society [3]. The IVD is found between the vertebrae and consists of the endplate, nucleus pulposus (NP), and annulus fibrosus (AF) [4,5,6,7]. Two factors contribute to IVD degeneration. First, the soft core of the disc (or NP) is composed mostly of water, and its water content gradually decreases as it ages; as a result, the disk thins, and shock absorption is impaired. The disc may also have damage resulting from cracks caused by injury. In particular, when the outer wall (or AF) of the disc is cracked, the disc expands and compresses the spinal nerve, causing great pain [2,8,9]. This low back pain causes difficulties in daily life, and as symptoms intensify over time, some activities may become impossible [5]. Therefore, if a patient is not treated in time, the symptoms may worsen, and the situation may be aggravated [10]. For this reason, clinicians must apply the treatment method most suitable for the patient’s condition by accurately diagnosing the severity of the IVD degeneration. Surgical therapy for patients with severe IVD degeneration has many side effects [11]. As such, conservative therapy, which is possible if an accurate diagnosis is performed before the degeneration has excessively progressed, can improve quality of life [5].

Currently, the main clinical method for diagnosing degenerative discs is magnetic resonance imaging, through which changes in water content can be detected. However, measuring matrix changes is difficult in the early stages of IVD degeneration, when conservative therapy can still be performed. Methods such as lumbar radiography and computed tomography are also used, but these are limited as definitive methods for diagnosing pain [12].

Cyclooxygenases (COXs) are the rate-limiting enzymes for the synthesis of prostaglandin E2, a major inflammatory mediator. COX-1 is expressed in most tissues, but COX-2 is known to be expressed only in specific biological conditions and environments [13]. In addition, COX-2 is closely involved in inflammation and is a mediator of pain conduction [14]. Recent research has indicated a correlation between COX-2 expression and the severity of disc degeneration [15]. One study showed that the inhibition of COX-2 induces the inhibition of vertebral endplate ossification, relieving pain in early stages of IVD degeneration [15]. Another study demonstrated the role of COX-2 as a biomarker for IVD degeneration by reporting variations in the number of COX-2–positive cells according to the level of IVD degeneration (based on a 1–5 scale according to the Pfirrmann classification system) in human specimens [16]. Given these findings, we attempted to develop a fluorescent probe capable of selectively binding to COX-2. Via this marker, the fluorescence intensity can vary according to COX-2 expression, indicating the degree of IVD degeneration and thereby suggesting a potential new diagnostic marker for degenerative discs.

Antibody-based immunofluorescence is typically used to identify specific cells or biomolecules. However, this method is limited in that the cells must be fixed or killed, and not living [17]. To date, most methods of visual identification of COX-2 in IVD-related studies have employed immunofluorescence [18,19,20,21,22]. Therefore, we utilized a method involving fluorescent molecules in this study. The goal was to develop diagnostic markers applicable to future cases by using small molecules able to penetrate living cells. Thus, we could demonstrate the correlation between the degree of COX-2 expression and IVD degeneration through molecular luminescence, and the findings can contribute to the understanding of pathogenesis and the development of treatments for IVD degeneration in the future.

## 2. Materials and Methods

### 2.1. Materials and Characterization

Information regarding the materials, synthesis, and other details required to generate the fluorescent markers is included in the Supporting Information section (Scheme S1, Table S1, and Figures S1–S25). The initial synthesis was performed based on a method detailed in previous research [23].

### 2.2. Spectroscopic Measurements

Absorption spectra were recorded on an OPTIZEN POP UV-Vis spectrophotometer (K Lab Co., Ltd., Daejeon, Republic of Korea), and fluorescence spectra were obtained with a FluoroMate FS-2 fluorescence spectrophotometer (SCINCO, Seoul, Republic of Korea) with a 1 cm standard quartz cell. The fluorescence quantum yield was determined using coumarin 307 (Φ = 0.95 in methanol) as described previously [24] (Figure S2 and Table S1).

### 2.3. Cell Culture

Human nucleus pulposus cells (HNPC; #4800; Science Cell, San Diego, CA, USA) were cultured in nucleus pulposus cell medium (#4801; Science Cell) supplemented with 10% fetal bovine serum (FBS; #0010; Science Cell), 1% nucleus pulposus cell growth supplement (NPCGS; #4852; Science Cell) and 1% penicillin–streptomycin solution (10,000 units/mL; P/S; #0503; Science Cell). Cells were maintained in a 5% CO_2_ incubator at 37 °C. Subculturing was performed using 0.05% trypsin/EDTA, which was prepared in dilution of Dulbecco’s Phosphate-Buffered Saline (DPBS; #0303; Science Cell) and 0.25% trypsin/EDTA × 1 (T/E; #0103; Science Cell) when the cells were more than 90% confluent, and samples were centrifuged at 1300 rpm for 10 min. 

HeLa cells (KCLB #10002; Korean Cell Line Bank, Seoul, Republic of Korea) were cultured in Dulbecco’s modified Eagle medium (DMEM; WelGene, Gyeongsan, Republic of Korea) supplemented with 10% fetal bovine serum and 1% penicillin–streptomycin solution (10,000 units/mL, SV30010, HyClone, Cytiva, Marlborough, MA, USA). Cells were maintained in a 5% CO_2_ incubator at 37 °C. Subculturing was performed using 0.25% trypsin/EDTA ×1 (#LS 015-10; WelGene) when the cells were more than 90% confluent, and samples were centrifuged at 1300 rpm for 10 min. 

The cells were treated and incubated with 2 μM **IBPC1** at 37 °C under 5% CO^2^ for 30 min. The cells were washed three times with phosphate-buffered saline (PBS) (Gibco) and then imaged after further incubation in a colorless serum-free medium for 30 min.

### 2.4. Cell Viability Assay

For the cell viability assessment, HeLa cells were seeded at approximately 1 × 10^4^ cells/well and cultured in a 96-well plate (#ISO 13485; SPL Life Sciences, Pocheon, Republic of Korea). Twenty-four hours after seeding, solutions of **IBPC1** were generated at concentrations of 2, 5, 10, 20, and 40 μM in Dulbecco phosphate-buffered saline, and 10 μL of each was added to the well plate and treated for 10 h. Then, an additional 10 μL per well of solution was added from a cell-counting kit-8 assay kit (#CK04-13; Dojindo, Kumamoto, Japan) and incubated for 2 h in a 5% CO_2_ incubator at 37 °C. To quantify cell viability, optical density was measured at 450 nm using a microplate reader (#1426305; Thermo Fisher Scientific, Waltham, MA, USA) (Figure S5).

### 2.5. Confocal Microscope Imaging

Cells were seeded in a confocal imaging dish (#101350; SPL Life Sciences) for live cell imaging. The experiment was conducted after 24 h of cell seeding; cells were treated with 1 mM COX-2 inhibitor (#236011; Sigma-Aldrich, St. Louis, MA, USA) for 1 h and with each concentration of LPS (#L2630; Sigma-Aldrich) along with 50 ng/mL interferon-γ (IFN-γ, #G305; Sigma-Aldrich) for 6 h. The experimental groups included the control, LPS (50 ng/mL) + IFN-γ, LPS (250 ng/mL) + IFN-γ, LPS (500 ng/mL) + IFN-γ, and LPS (500 ng/mL) + IFN-γ + COX-2 inhibitor. All cells were washed with DMEM twice and stained with 2 μM **IBPC1** for 30 min in imaging dishes. After this procedure, the fluorescence spectrum and photostability of intracellular **IBPC1** were measured (Figures S3 and S6). Normal and damaged rat disc tissues were stained with 10 μM **IBPC1** for 1 h and then imaged. The images of **IBPC1**-labeled cells and tissues were obtained using a Leica TCS SP5II laser scanning confocal microscope (Leica Microsystems GmbH, Wetzlar, Germany) with a 405 nm diode laser for excitation and using a 40× oil objective lens (numerical aperture (NA) = 1.30). As for the fluorescence spectra, the lambda scans performed spectra between 400 and 600 nm with 5 nm bin widths with a 405 nm diode laser for excitation. The fluorescence images were captured at the 400–600 nm emission filter level with a 405 nm diode laser for excitation. The obtained lambda stacks and average fluorescence intensity of individual cells were analyzed with Leica LAS AF Lite (Leica Microsystems GmbH) software.

### 2.6. Western Blot

Cells were lysed on ice in PRO-PREP protein extraction solution (iNtRON Biotechnology, Seongnam, Republic of Korea). The protein concentration was analyzed by Pierce BCA protein assay kit (Thermo Scientific). The protein samples (30 μg/lane) were resolved using SDS-PAGE and transferred to nitrocellulose membranes (Bio-Rad, Hercules, CA, USA). The membranes were blocked in 5% skim milk in TBST (10 mM Tris–HCl, pH 8.0, 150 mM NaCl, 0.05% Tween 20) for 1 h and incubated with the specific primary antibody in the blocking solution at 4 °C overnight. Antibodies against COX-1 (#sc-19998; Santa Cruz Biotechnology, Dallas, TX, USA), COX-2 (#12282; Cell Signaling Technology, Danvers, MA, USA), and GAPDH (#sc-47724; Santa Cruz Biotechnology) were used. The secondary antibodies used were goat anti-mouse IgG (#ADI-SAB-100-J; Enzo, Farmingdale, NY, USA) or goat anti-rabbit IgG (#ADI-SAB-300-J; Enzo). Finally, the detection was performed using an enhanced chemiluminescence system (Amersham Pharmacia Biotech, Piscataway, NJ, USA).

### 2.7. Animal Experimental Procedure and Tail-Puncture IVD Degeneration Modeling

All animal experiments were approved by the Institutional Animal Care and Use Committee (IACUC220190) at the CHA University School of Medicine. The animals were 10-week-old female Sprague–Dawley rats (220–250 g) supplied by Korean Animal Technology (Koatech, Pyeongtaek, Republic of Korea). Before surgery, the animals were fed and acclimatized for 1 week to reduce the effects of stress. Constant temperature, humidity, and photoperiod were maintained at the animal facility (humidity, 55–65%; temperature, 24 °C ± 3 °C; photoperiod, 12 h light/12 h dark). Before modeling surgery, all rats were anesthetized with a 1:1:1 mixture of zolazepam (Zoletil, 50 mg/kg, intraperitoneal; Virbac, Carros, France), tiletamine, and xylazine (Rompun, 10 mg/kg, intraperitoneal; Bayer, Seoul, Republic of Korea). The coccygeal vertebrae of the anesthetized rats were disinfected with iodine and ethanol to prepare for surgery. A blade was used to make an incision at Co5-7, the disc was exposed between the tail muscles, and the center of the disc was punched with a needle. After the inserted needle was rotated 360°, it was left in place for 30 s. Moderate IVD degeneration was modeled with a 26G needle at the disc between Co6 and Co7, whereas severe degeneration was modeled with an 18G needle between Co5 and Co6. Specifically, an incision at Co4-5 was used for the control group, insertion of an 18G needle at Co5-6 represented severe injury, and insertion of a 26G needle at Co6-7 represented moderate injury [25,26,27]. After suture, the incision site was disinfected with iodine, and the animal recovered on a heating pad at 39 °C.

### 2.8. Tissue Processing and Sectioning

Three days after the induction of IVD degeneration, all rats were sacrificed. We obtained coccygeal vertebrae discs (Co4-5, Co5-6, and Co6-7) from the target rat tail. The coccygeal vertebrae were decalcified in RapidCal Immuno (BBC Biochemical, Mount Vernon, WA, USA) and then sequentially dehydrated with 70%, 80%, 90%, 95%, and 100% ethanol. The dehydrated tissue was cleared in xylene and then embedded in paraffin wax. The paraffin block was prepared by pouring the prepared paraffin solution on the tissue. Finally, using a microtome (#RM2255; Leica, Wetzlar, Germany), a tissue sample with an axial thickness of 5 μm was prepared and placed on a glass slide.

### 2.9. Fluorescent Probe Staining and Immunofluorescence

All tissue samples on the slides were stained and tested using the following method. Before staining, samples were deparaffinized using xylene. Antigen retrieval was performed with a pepsin solution (#E06-50; GBI Labs, Bothell, WA, USA) at 37 °C for 10 min after ethanol dehydration (99.9%, 95%). **IBPC1** staining was performed with DAPI (#D1306; Invitrogen, Waltham, MA, USA). Samples were pretreated with DAPI for 10 min, washed with 0.1% 1× PBS-Tween (#P9416; Sigma-Aldrich), then stained in an incubator for 2 h with 20 μM **IBPC1** dissolved in Dulbecco phosphate-buffered saline. For immunofluorescence, the tissue was stained with 1:400 COX-2 primary antibody (#12282; Cell Signaling Technology, Danvers, MA, USA) for 1.5 h at 24 °C ± 3 °C after antigen retrieval. Then, the tissue was washed and stained with 1:200 goat anti-rabbit IgG secondary antibody Alexa Fluor 488 (#A-11008; Invitrogen) for 50 min at 24 °C ± 3 °C. After staining, samples were washed and dyed with DAPI for 10 min. Again, they were washed with PBS-Tween and mounted on the cover glass. Immunofluorescence results after **IBPC1** staining were obtained via imaging with a digital slide scanner (ZEISS Axio Scan.Z1; Zeiss, Oberkochen, Germany), and analysis was performed using the Zen 3.1 Blue edition software program (Zeiss). For imaging, the slide scanner was set at an FL ×20 mode, the auto-focus function was used to determine the imaging area based on DAPI, and the final image was acquired by setting the value corresponding to Alexa Fluor 488.

## 3. Results and Discussion

### 3.1. Design and Synthesis of the COX-2–Selective Fluorescence Probe (IBPC1)

**Figure 1:**
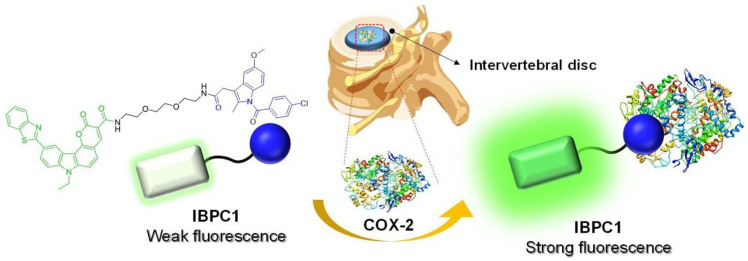
Strategy for evaluation of intervertebral disc degeneration using the cyclooxygenase-2 (COX-2)-selective fluorescent probe indomethacin-adopted benzothiazole–pyranocarbazole-1 (**IBPC1**).

We developed a new indomethacin-adopted fluorescence probe, **IBPC1**, for the detection of COX-2 in living cells and IVD tissues (Figure 1). The COX-2-selective probes in which the carboxylic acid groups of indomethacin are substituted with amide or ester groups have been reported for COX-2 detection and inhibition [28,29,30,31,32,33]. The amide or ester groups of indomethacin eliminate COX-1 inhibitory activity while maintaining COX-2 inhibitory activity [34]. The fluorophore (benzothiazole-pyranocarbazole 1 [**BPC1**]) was designed as a dipolar structure containing an electron-donor–π–electron-acceptor system. In addition, the pyranocarbazole provides a rigid core and elongated conjugation system. Indomethacin, a classic COX-2 inhibitor, was introduced for the detection of COX-2 [35]. **BPC1** was linked to indomethacin using 1,2-bis(2-aminoethoxy)ethane for the linker. Detailed synthesis methods for **IBPC1** and each intermediate are described in Scheme S1.

### 3.2. Photophysical Properties of IBPC1

**Figure 2:**
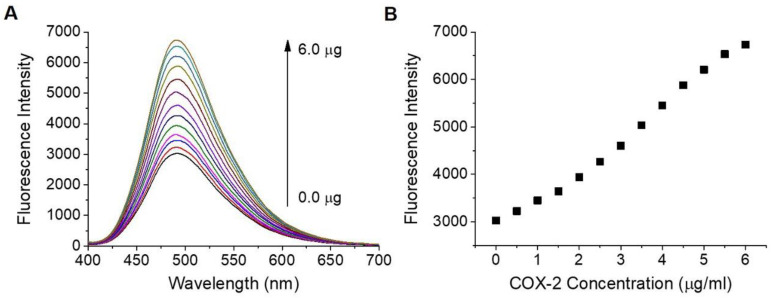
(**A**) Changes in fluorescence spectrum of **IBPC1** according to the concentration of cyclooxygenase-2 (COX-2) (0–6 μg/mL). (**B**) Plot of fluorescence intensity against COX-2 concentration for **IBPC1** in ethanol: phosphate-buffered saline (1:1 *v*/*v*) solution. The excitation wavelength was 388 nm.

The water solubility of **IBPC1** was approximately 2 μM in phosphate-buffered saline (PBS, pH 7.4) (Figure S1). **IBPC1** shows an absorption maximum (λ_abs_) at 378 nm (ε = 0.70 × 10^−4^ M^−1^ cm^−1^) and a fluorescence maximum at 505 nm (Φ = 0.02) in PBS buffer (Figure S2 and Table S1, respectively). The fluorescence spectrum of **IBPC1** showed a solvatochromic shift as the solvent polarity was increased in the order of 1,4-dioxane < EtOH < EtOH: PBS (1:1 *v/v*) < PBS buffer, and the fluorescence quantum yield (Φ) increased as the solvent polarity decreased (Figure S2 and Table S1). The solvatochromic shifts with increasing solvent polarity indicated the utility of **IBPC1** as an environment-sensitive probe, and these results allowed us to assess the **IBPC1** environment in the cells. Moreover, the emission spectrum (~470 nm) from the **IBPC1**-labeled cells resembles that measured in EtOH (Figure S3 and Table S1), suggesting that this solvent can adequately represent cell polarity. Next, we demonstrated the fluorescence response of **IBPC1** to COX-2 in EtOH: PBS (1:1 *v/v*) solution. As shown in Figure 2, after the addition of COX-2, the fluorescence intensity of **IBPC1** gradually increased with an increasing amount of COX-2 at 491 nm. However, **IBPC1**-negligible changes in response to COX-1 (Figure S4). Moreover, **IBPC1** is insensitive to pH in the biologically relevant pH range (Figure S5). This result suggests that the **IBPC1** that we developed can work well in various pH environments in vivo.

### 3.3. Evaluation of COX-2 Detection Ability in Cells

Before the full-scale cell experiment, a separate experiment was conducted to determine the degree of cytotoxicity of **IBPC1** in living cells. We observed cell viability while incubating **IBPC1** with nucleus pulposus cells (NPCs) in the middle of the intervertebral disc for 10 h at concentrations of 2–40 μM. Compared to the control condition, even at up to 40 μM, we observed almost 80% viability and very little cytotoxicity (Figure S6A). Additionally, we examined cell viability while staining 10 μM of **IBPC1** with NPCs for 2, 6, 12, 18, and 24 h. Compared to the control condition, even at up to 24 h, we observed almost 80% viability and low toxicity (Figure S6B). Based on these results, we proceeded with the experiment at 2 μM, at which cell viability was 90% or greater.

Subsequently, experiments were conducted to establish the basic distribution of **IBPC1** in cells and the optimal conditions for imaging. After staining NPCs with 2 μM of **IBPC1** for 30 min, the fluorescence by COX-2 was observed in NPCs (Figure S3A). In addition, the fluorescence spectrum of **IBPC1** in the intracellular environment was analyzed using Leica confocal data analysis software. Fluorescence was observed over 400–700 nm, and the maximum emission wavelength was confirmed to appear at approximately 470 nm (Figure S3B). Based on these results, we established a 400–600 nm range to compare and analyze the fluorescence intensity with as much sensitivity as possible in the subsequent cell- and tissue-imaging experiments. In addition, we experimented to verify the photostability of **IBPC1** following long-term light exposure. After staining NPCs with 2 μM **IBPC1** for 30 min, changes in fluorescence intensity were observed in response to an irradiating laser used at 2 s intervals for 2000 s. Fluorescence was maintained at approximately 85% or higher relative to the initial level (Figure S7). This suggests that **IBPC1** is observable for a long duration in living cells. To confirm **IBPC1** has selectivity for COX-2 in NPCs, we conducted co-localization experiments co-labeled with **IBPC1** and the COX-2 antibody for the detection of COX-2. The fluorescence images of **IBPC1** and COX-2 antibody overlapped well with the Pearson’s co-localization coefficient value of 0.85 (Figure S8).

**Figure 3:**
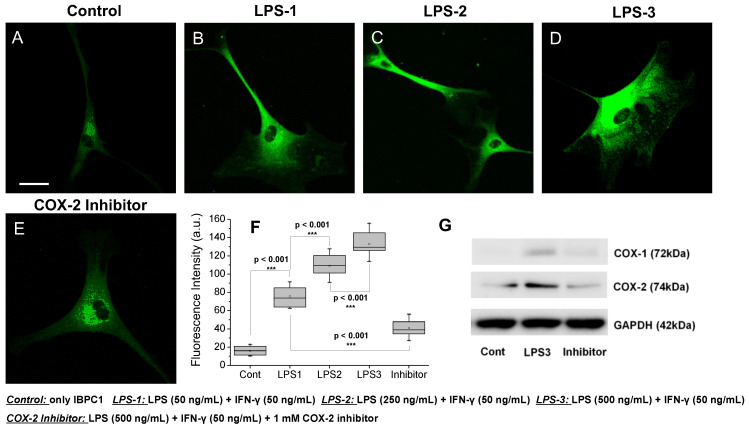
(**A**–**E**) Fluorescence images of **IBPC1** by interaction with COX-2 in nucleus pulposus cells (NPCs). Cells were labeled with 2 μM **IBPC1** (**A**) and pretreated with (**B**) 50, (**C**) 250, (**D**) 500 ng/mL LPS, and 50 ng/mL IFN-γ for 5 h. (**E**) After pretreatment with 500 ng/mL LPS and 50 ng/mL IFN-γ for 5 h, 1 mM COX-2 inhibitor was subsequently treated for 1 h. (**F**) Box plot of fluorescence intensity in (**A**–**E**). (**G**) Western blot analysis of COX-1 and COX-2. The band intensities were normalized to the respective GAPDH band. The fluorescence intensities were collected at 400–600 nm upon excitation at 405 nm and measured in 500 randomly chosen regions. Asterisks indicate statistical significance (*** *p* < 0.001). Cells shown are representative images from replicate experiments (*n* = 5). Scale bars: (**A**–**E**) 60 μm.

Finally, experiments were conducted to determine whether **IBPC1** was selective for COX-2 even in living cells and whether it exhibited varying fluorescence intensity depending on the level of COX-2 expression. As reported in several recent studies, COX-2 expression is increased by inflammatory stimuli [16,36]. To confirm that COX-1 and COX-2 expression is increased by inflammatory stimuli, we performed qPCR to analyze COX-1 and COX-2 expression in pretreatment of NPCs, as COX-1 and COX-2 expression varies according to the concentrations of LPS and IFN-γ, which are known inducers of inflammation (Figure S9). When NPCs were pretreated, LPS (50 ng/mL) and IFN-γ (50 ng/mL) showed an increased COX-2 expression level. However, cells pretreated with an LPS (250 ng/mL) showed negligible changes compared with those pretreated with an LPS (50 ng/mL), and there was no statistical difference. The group pretreated with 500 ng/mL LPS showed a significantly increased expression level, and the experimental group pretreated with the 1 mM COX-2 inhibitor confirmed that the expression level of COX-2 was significantly lowered (Figure S9B). However, the expression level of COX-1 showed almost negligible changes (Figure S9A). We attempted to observe the expression of COX-2 along with the corresponding fluorescence intensity in cells. When the NPCs were pretreated with LPS (50 ng/mL) and IFN-γ (50 ng/mL), a 4.8-fold increase in fluorescence intensity was observed compared to cells treated only with **IBPC1** (Figure 3B,F). Cells pretreated with a higher LPS concentration of 250 and 500 ng/mL showed a significant 6.9- and 8.4-fold increase in fluorescence intensity compared to the control condition (Figure 3C,D,F). In contrast, in the experimental group pretreated with the 1 mM COX-2 inhibitor, no significant difference was observed from the fluorescence intensity of the control group, even though LPS and IFN-γ were administered together (Figure 3E,F). Similar results were observed with HeLa cells (Figure S10). As is well known from the past, in order to show the results of cancer cells showing a relatively high COX-2 expression level compared to normal cells as a control group, the results showing the expression level in HeLa cells are shown in Figure S10. In the result, the HeLa cell control showed an average fluorescence value of ~31, whereas the NPCs showed a fluorescence value of ~17 (Figure 3F and Figure S10F). Furthermore, we investigated COX-1 and COX-2 expression levels by Western blot analysis to confirm that COX-2 expression is increased by inflammatory stimuli and **IBPC1** has selectivity for COX-2. As shown in the Western blot analysis results (Figure 3G), the COX-2 expression level was increased in NPCs pretreated with LPS (500 ng/mL) and IFN-γ (50 ng/mL) compared with only stained **IBPC1** (control group). These results suggest that **IBPC1** not only represents the expression level of COX-2 through the observed fluorescence intensity but also has excellent selectivity for COX-2 considering the results of the COX-2 inhibitor.

### 3.4. Efficacy of IBPC1 in an Animal Model of IVD Degeneration

**Figure 4:**
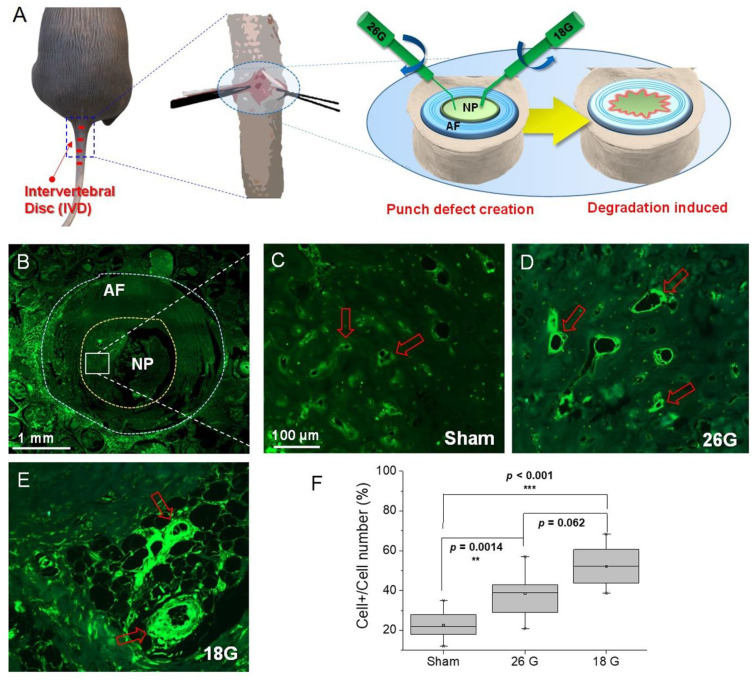
(**A**) Schematic diagram showing the induction of a rat intervertebral disc (IVD) injury model. (**B**–**E**) Immunofluorescence images of rat intervertebral disc with COX-2 primary antibody. (**B**) Low-magnification image showing COX-2 distribution in a normal intervertebral disc. (**C**–**E**) High-magnification images showing COX-2 distribution in (**C**) normal and (**D**) 26G- and (**E**) 18G-needle-puncture-injured intervertebral discs. Red arrows point to representative cells showing differences before and after overexpression. (**F**) Box plot of fluorescence intensity in (**B**–**E**). The fluorescence images and intensities were acquired by using a slide scanner (Zeiss Axio Scan. Z1, ^®^Carl Zeiss) and measured in 100 randomly chosen regions. Asterisks indicate statistical significance (** *p* < 0.01, *** *p* < 0.001). The image above is a representative image from 7 repeated experiments. Scale bars: (**B**) 1 mm and (**C**–**E**) 100 μm.

Inflammation is known to be a primary cause of IVD degeneration, and recent reports indicate that greater COX-2 expression is found in more advanced cases of IVD degeneration [16,37]. To reproduce this phenomenon in an animal model, we constructed an IVD degeneration model using rats. The experiment was designed to represent varying degrees of inflammation in the NP area according to the size of the experimental needle used for IVD injury (Figure 4A). First, to confirm the difference in COX-2 expression according to the degree of inflammation, immunofluorescence was performed using a COX-2 antibody. Figure 4B is a low-magnification image of the NP and surrounding AF of the rat’s caudal IVD, whereas Figure 4C–E are high-magnification images of the NP area. We observed that as the needle size was increased from 26G to 18G, inflammation was strongly induced, and the fluorescence indicated by COX-2 was similarly strong. The qualitative analysis of this result was derived as shown in Figure 4F. The expression of COX-2 in response to injury from varying needle sizes was analyzed by cell number percentage (%). The expression level was observed to be 23% ± 12% in the sham group, 38% ± 14% in the 26G (moderate injury) group, and 52% ± 16% in the 18G (severe injury) group. These results indicate that the animal model that we established is appropriate for confirming the difference in the expression level of COX-2 according to the degree of inflammation.

**Figure 5:**
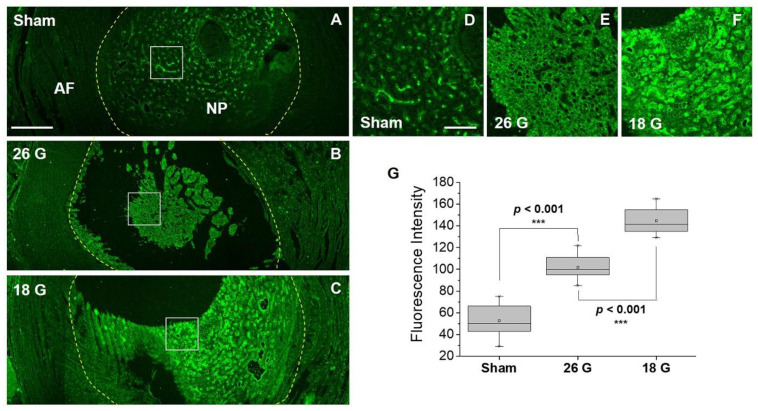
Fluorescence confocal microscopy images showing NP (nucleus pulposus) and AF (annulus fibrosus) of rat intervertebral discs by 10 µM **IBPC1**. (**A**–**C**) Fluorescence images according to COX-2 distribution in intervertebral disc tissue under (**A**) sham, (**B**) 26G-, and (**C**) 18G-needle-puncture-damaged conditions, respectively. (**D**–**F**) Magnified images of the areas indicated by the white squares in **A**–**C**. (**G**) Box plot was obtained based on the fluorescence values of the NP region shown in images (**D**–**F**). The fluorescence images were obtained at 400–600 nm upon excitation at 405 nm and acquired at 100 randomly chosen regions. Asterisks indicate statistical significance (*** *p* < 0.001). Disc tissues shown are representative images from replicate experiments (*n* = 7). Scale bars: (**A**–**C**) 500 and (**D**–**F**) 100 μm.

Next, we applied **IBPC1** to the IVD degeneration model rat tissue established in the previous experiment. Figure 5A–C represent low-magnification confocal fluorescence images of **IBPC1** which include fluorescence based on the interaction with COX-2. In tissues damaged by the 26G and 18G needles, as inflammation was induced, the appearance of the damaged NP was observed visually compared to that of the sham NP, and higher fluorescence intensity was also observed (Figure 5B,C). We performed a precise analysis by selectively enlarging the NP region where COX-2 expression primarily appears. The fluorescence intensity was the greatest in the 18G-needle-punched group, which corresponded to relatively severe injury (Figure 5D–F). We compared and analyzed these results qualitatively with box plots. The fluorescence intensity was measured at 53 ± 22 in the sham group, 102 ± 20 in the 26G (moderate injury) group, and 145 ± 19 in the 18G (severe injury) group (Figure 5G).

These findings resemble the trend in the results obtained using the fluorescent COX-2 antibody. However, compared to the 23% to 52% comparison in the immunofluorescence analysis, the difference between the experimental groups (from 53 to 145) can be more clearly distinguished in the fluorescence intensity analysis using **IBPC1**. The method involving the fluorescent molecular compound **IBPC1** has the advantage of being an imaging method that can be used in living cells or tissues, in addition to being a much easier and faster process. These results suggest that **IBPC1** can be widely used in future studies of IVD degeneration.

## 4. Conclusions

In conclusion, **IBPC1** exhibits varying fluorescence intensity depending on the level of expression of COX-2, a biomolecule known to strongly relate to IVD degeneration. **IBPC1** was synthesized by combining indomethacin, which is known to have COX-2 targeting ability, with benzothiazole-pyranocarbazole, which shows strong fluorescence at approximately 500 nm. Furthermore, **IBPC1** exhibited high fluorescence intensity when inflammation was strongly induced in living cells, whereas fluorescence was lower in the cells pretreated with an inhibitor, demonstrating selectivity for COX-2. In addition, **IBPC1** allowed us to clearly distinguish the difference in the degree of COX-2 expression due to the induction of inflammation in rat disc tissue in which IVD degeneration was induced. These results provide insight into the detection of COX-2 expression in IVD degeneration tissue and indicate that **IBPC1** can contribute to future diagnosis and treatment studies of IVD degeneration.

## Data Availability

The original contributions presented in the study are included in the article and the Supplementary Materials. Further inquiries can be directed to the corresponding authors.

## Supplementary Materials

The following supporting information can be downloaded at: https://www.mdpi.com/article/10.3390/jfb14040192/s1, Scheme S1. Synthesis of **IBPC1**; Figure S1. (A) Changes in fluorescence spectrum according to the concentration of **IBPC1**. (B) Plot of fluorescence intensity against concentration for **IBPC1** in phosphate buffer. The excitation wavelength was 380 nm. Figure S2. Normalized emission spectra of **IBPC1** in 1,4-dioxane, EtOH, EtOH: PBS (*v/v*, 1:1) and PBS buffer; Figure S3. (A) Fluorescence image of 2 µM **IBPC1** in nucleus pulposus cells (NPCs). Scale bar: 60 µm. (B) Fluorescence spectra of **IBPC1** in NP cells. The excitation wavelengths were 405 nm. Figure S4. Changes in fluorescence spectrum of **IBPC1** according to the concentration of cyclooxygenase-1 (COX-1) (0–4 μg/mL). Figure S5. Effect of pH (4.0–9.0) on the fluorescence intensity of 2 μM **IBPC1** in universal buffer (0.1 M citric acid, 0.1 M KH_2_PO_4_, 0.1 M Na_2_B_4_O_7_, 0.1 M Tris, 0.1 M KCl). The excitation wavelength was 380 nm. Figure S6. Viability of nucleus pulposus cells (NPCs) in the presence of **IBPC1,** as measured using a Cell Counting Kit-8 assay kit. The cells were incubated with (A) 0–40 μM **IBPC1** for 10 h and (B) 10 μM **IBPC1** for 2, 6, 12, 18, and 24 h. Five independent experiments were performed. Figure S7. (A) Confocal fluorescence images of **IBPC1**-labeled (2 μM) nucleus pulposus cells (NPCs). (B) The relative fluorescence intensity from A to C in (A) as a function of time. Cells shown are representative images from replicate experiments (*n* = 5). Figure S8. Co-localization fluorescence images of nucleus pulposus cells (NPCs) incubated with (A) **IBPC1** (5 μM) for 30 min and then with (B) COX-2 antibody for 1 day at 4 °C. (C) The merged image and the Pearson correlation coefficient were calculated accordingly. Scale bars: 60 μm. Figure S9. qPCR analysis of nucleus pulposus cells (NPCs) that were pretreated with LPS. Relative mRNA expression levels of (A) COX-1 and (B) COX-2 were normalized against the corresponding levels of 18s rRNA. Asterisks indicate statistical significance (*** *p* < 0.001). The cells shown are representative images from replicate experiments (*n* = 5). Figure S10. (A–E) Fluorescence images of **IBPC1** by interaction with COX-2 in HeLa cells. Cells were labeled with 2 μM **IBPC1** (A) and pretreated with (B) 50, (C) 250, (D) 500 ng/mL LPS, 50 ng/mL IFN-γ for 5 h. (E) 1 mM COX-2 inhibitor was treated for 1 h after being pretreated with 500 ng/mL LPS, 50 ng/mL IFN-γ for 5 h. (F) Box plot of fluorescence intensity in A–E. The fluorescent intensities were collected at 400–600 nm upon excitation at 405 nm and measured in 500 randomly chosen regions. Asterisks indicate statistical significance (****p* < 0.001). Cells shown are representative images from replicate experiments (*n* = 5). Scale bars: (a–e) 60 μm. Figures S11–S24. ^1^H-NMR and ^13^C-NMR spectrum. Figure S25. HRMS spectrum of **IBPC1**. Table S1. Photophysical properties of **IBPC1** in various solvents.
