# Supplementary material for: Development of a COX-2-Selective Fluorescent Probe for the Observation of Early Intervertebral Disc Degeneration
Source: J Funct Biomater. 2023 Mar 29;14(4):192. doi: 10.3390/jfb14040192 (PMC10146728; doi:10.3390/jfb14040192)
Supplement: Supplementary file 1 [file jfb-14-00192-s001.zip › jfb-2173165-supplementary.pdf]

Supporting Information to Accompany

Development of a COX-2-Selective Fluorescent Probe for the Observation of Early Intervertebral Disc Degeneration

Cheol Ho Heo [1,2,†], Eun Ji Roh [3,4,†], Jaehee Kim [2], Hyemin Choi [3], Ho Yeon Jang [1], Giseong Lee [2,5,*], Chang Su Lim [3,*] and Inbo Han [3,*]

[1] Department of Applied Chemistry, Kookmin University, Seoul 02707, Republic of Korea
[2] Pure Chem Co., Ltd., Knu Start-up CUBE, Chunchenon 24341, Republic of Korea
[3] Department of Neurosurgery, CHA University School of Medicine, CHA Bundang Medical Center, Seongnam-si 13496, Republic of Korea
[4] Department of Biomedical Science, CHA University School of Medicine, CHA Bundang Medical Center, Seongnam-si 13496, Republic of Korea
[5] College of General Education, Kookmin University, Seoul 02707, Republic of Korea
* Correspondence: gsrecall@kookmin.ac.kr (G.L.); Chang Su Lim, cslim8112@gmail.com (C.S.L.); hanib@cha.ac.kr (I.H.)
† These authors contributed equally to this work and share first authorship.

Table of Contents

	Page
**Synthesis of IBPC1**..	S3
**Water solubility**..	S6
**Cell viability**..	S9
**Photostability**..	S9
**Co-localization experiment**..	S10
**Quantitative real-time PCR (qPCR)**	S11
**Figure S1**. (A) Changes in fluorescence spectrum according to the concentration of **IBPC1**. (B) Plot of fluorescence intensity against concentration for **IBPC1** in phosphate buffer. The excitation wavelength was 380 nm..	S6
**Figure S2.** Normalized emission spectra of **IBPC1** in 1,4-dioxane, EtOH, EtOH: PBS (v/v, 1:1) and PBS buffer..	S6
**Figure S3.** (A) Fluorescence image of 2 μM **IBPC1** in nucleus pulposus cells (NPCs). Scale bar: 60 μm. (B) Fluorescence spectra of **IBPC1** in NP cells. The excitation wavelengths were 405 nm..	S7
**Figure S4**. Changes in fluorescence spectrum of **IBPC1** according to the concentration of cyclooxygenase-1 (COX-1) (0-4 μg/mL)..	S8
**Figure S5.** Effect of pH (4.0-9.0) on the fluorescence intensity of 2 μM **IBPC1** in universal buffer (0.1 M citric acid, 0.1 M KH_2PO_4, 0.1 M $Na_2B_4O_7$, 0.1 M Tris, 0.1 M KCl). The excitation wavelength was 380 nm..	S8
**Figure S6.** Viability of nucleus pulposus cells (NPCs) in the presence of **IBPC1** as measured using a Cell Counting Kit-8 assay kit. The cells were incubated with (A) 0-40 μM **IBPC1** for 10 h and (B) 10 μM.**IBPC1** for 2, 6, 12, 18, and 24 h. Five independent experiments were performed..	S9
**Figure S7.** (A) Confocal fluorescence images of **IBPC1**-labeled (2 μM) nucleus pulposus cells (NPCs). (B) The relative fluorescence intensity from A-C in (A) as a function of time. Cells shown are representative images from replicate experiments (n = 5)..	S9
**Figure S8.** Co-localization fluorescence images of nucleus pulposus cells (NPCs) incubated with (A) **IBPC1** (5 μM) for 30 min and then with (B) COX-2 antibody for 1 day at 4 ºC. (C) The merged image and the Pearson correlation coefficient calculated accordingly. Scale bars: 60 μm..	S10

Figure S9. qPCR analysis of nucleus pulposus cells (NPCs) that were pretreated with LPS. Relative mRNA expression levels of (A) COX-1 and (B) COX-2 were normalized against the corresponding levels of 18s rRNA. Asterisks indicate statistical significance (***p < 0.001). The cells shown are representative images from replicate experiments (n = 5)……… S11

Figure S10. (A-E) Fluorescence images of **IBPC1** by interaction with COX-2 in HeLa cells. Cells were labeled with 2 μM **IBPC1** (A) and pretreated with (B) 50, (C) 250, (D) 500 ng/mL LPS, 50 ng/mL IFN-γ for 5 hr. (E) 1 mM COX-2 inhibitor was treated for 1 hr after being pretreated with 500 ng/mL LPS, 50 ng/mL IFN-γ for 5 hr. (F) Box-plot of fluorescent intensity in A-E. The fluorescent intensities were collected at 400–600 nm upon excitation at 405 nm and measured in 500 randomly chosen regions. Asterisks indicate statistical significance (***p < 0.001). Cells shown are representative images from replicate experiments (n =5). Scale bars: (a-e) 60 μm………………………………………………. S12

Figure S11. ^{1}H-NMR spectrum (400 MHz) of **2** in DMSO………………………………….. S13
Figure S12. ^{13}C-NMR spectrum (400 MHz) of **2** in DMSO…………………………………. S13
Figure S13. ^{1}H-NMR spectrum (400 MHz) of **3** in DMSO………………………………….. S14
Figure S14. ^{13}C-NMR spectrum (400 MHz) of **3** in DMSO…………………………………. S14
Figure S15. ^{1}H-NMR spectrum (400 MHz) of **4** in DMSO………………………………….. S15
Figure S16. ^{13}C-NMR spectrum (100 MHz) of **4** in DMSO…………………………………. S15
Figure S17. ^{1}H-NMR spectrum (400 MHz) of **5** in $CDCl_3$………………………………... S16
Figure S18. ^{13}C-NMR spectrum (100 MHz) of **5** in $CDCl_3$………………………………… S16
Figure S19. ^{1}H-NMR spectrum (400 MHz) of **BPC1** in DMSO…………………………….. S17
Figure S20. ^{13}C-NMR spectrum (100 MHz) of **BPC1** in DMSO……………………………. S17
Figure S21. ^{1}H-NMR spectrum (400 MHz) of **8** in $CDCl_3$………………………………... S18
Figure S22. ^{13}C-NMR spectrum (400 MHz) of **8** in $CDCl_3$………………………………… S18
Figure S23. ^{1}H-NMR spectrum (400 MHz) of **IBPC1** in $CDCl_3$……………………………. S19
Figure S24. ^{13}C-NMR spectrum (100 MHz) of **IBPC1** in $CDCl_3$…………………………… S19
Figure S25. HRMS spectrum of **IBPC1**………………………..………………………… S20
Table S1. Photophysical properties of **IBPC1** in various solvents…………………………... S7

Synthesis of IBPC1. Compound **1** was prepared by the method described by Bhosale et al. (2012)[1], and compounds **6** and **7** were purchased from Sigma-Aldrich.

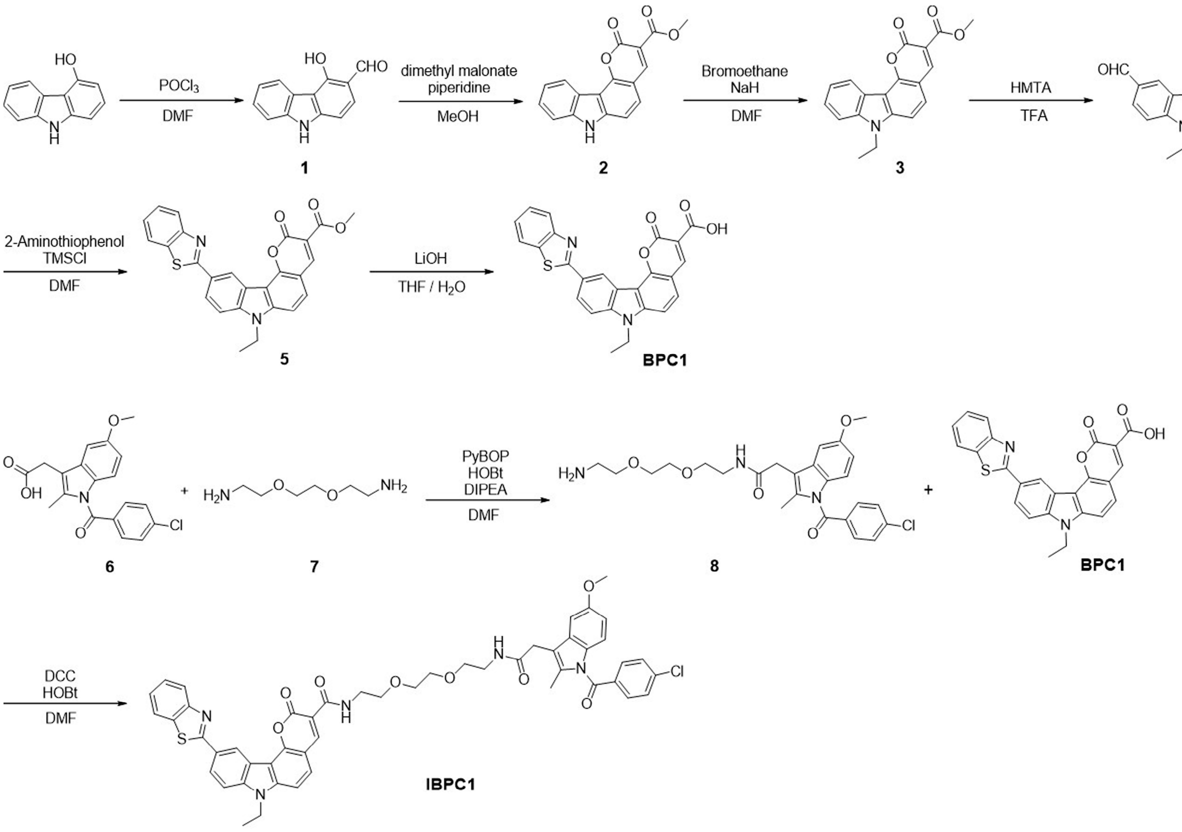

Scheme S1. Synthesis of **IBPC1**.

Methyl 2-oxo-2,7-dihydropyrano[3,2-*c*]carbazole-3-carboxylate (**2**). A mixture of **1** (600 mg, 2.84 mmol) and dimethylmalonate (750 mg, 5.68 mmol) in methanol (MeOH, 20 mL) was slowly added to piperidine (600 mg, 7.10 mmol), and the mixture was refluxed for 6 hours. The mixture was cooled, and the solvent was evaporated. The product was neutralized with acetic acid and extracted with dichloromethane (DCM). The organic layer was washed with H_2O and brine, dried over sodium sulfate (Na_2SO_4), and concentrated to obtain the crude product. The product was purified by recrystallization from MeOH. Yield: 78%; ^{1}H NMR (dimethyl sulfoxide [DMSO], 400 MHz): δ (ppm) 12.22 (s, 1H), 8.97 (s, 1H), 8.31 (d, J = 7.6 Hz, 1H), 7.88 (d, J = 8.4 Hz, 1H), 7.64 (d, J = 8.4 Hz, 1H), 7.53 (d, J = 8.4 Hz, 2H), 7.38 (t, J = 7.6 Hz, 1H), 3.85 (s, 3H); ^{13}C NMR (DMSO, 100 MHz): δ (ppm) 164.19, 156.90, 152.56, 151.64, 144.86, 139.95, 128.08, 126.92, 122.63, 121.35, 120.74, 112.40, 111.36, 110.05, 109.72, 109.30, 52.61.

Methyl 7-ethyl-2-oxo-2,7-dihydropyrano[3,2-*c*]carbazole-3-carboxylate (**3**). A solution of **2** (600 mg, 2.05 mmol) in dimethylformamide (DMF, 15 mL) was added to sodium hydride (NaH; 60% in mineral oil, 123 mg total, 74 mg as NaH, 3.07 mmol) at 0°C, and the mixture was stirred for 1 hour at room temperature. To this mixture, bromoethane (334 mg, 3.07 mmol) was added, and the solution was stirred for 6 hours. Then, the mixture was added to ammonium chloride (*aq*) and extracted with DCM. The organic layer was washed with H_2O and brine, dried over Na_2SO_4, and concentrated to obtain the crude product. The product was purified by flash column chromatography using

DCM/MeOH (98:2) as the eluent. Yield: 45%; ^{1}H NMR (DMSO, 400 MHz): δ (ppm) 8.96 (s, 1H), 8.34 (d, J = 7.6 Hz, 1H), 7.95 (d, J = 8.8 Hz, 1H), 7.80 (d, J = 8.0 Hz, 1H), 7.72 (d, J = 8.8 Hz, 1H), 7.60 (t, J = 8.0 Hz, 1H), 7.43 (t, J = 7.4 Hz, 1H), 4.57 (q, J = 7.2 Hz, 2H), 3.85 (s, 3H), 1.36 (t, J = 7.2 Hz, 3H); ^{13}C NMR (DMSO, 100 MHz): δ (ppm) 164.14, 156.81, 152.42, 151.52, 144.34, 139.89, 128.19, 127.02, 122.83, 121.64, 120.56, 111.52, 110.78, 110.20, 108.91, 107.92, 52.63, 38.22, 14.42.

Methyl 7-ethyl-10-formyl-2-oxo-2,7-dihydropyrano[3,2-*c*]carbazole-3-carboxylate (**4**). A solution of **2** (250 mg, 0.78 mmol) in trifluoroacetic acid was added to hexamethylenetetramine (218 mg, 1.56 mmol), and the mixture was stirred for 3 hours at room temperature. The solvent was evaporated, and the product was neutralized with sodium carbonate (*aq*) and extracted with DCM. The organic layer was washed with H_2O and brine, dried over Na_2SO_4, and concentrated to obtain the crude product. The product was purified by flash column chromatography using DCM/MeOH (98:2) as the eluent. Yield: 73%; ^{1}H NMR (DMSO, 400 MHz): δ (ppm) 10.16 (s, 1H), 8.93-9.00 (m, 1H), 8.76-8.80 (m, 1H), 8.11 (d, J = 8.4 Hz, 1H), 8.03 (d, J = 6.7 Hz, 1H), 7.97 (d, J = 8.4 Hz, 1H), 7.80 (d, J = 8.6 Hz, 1H), 4.62 (q, J = 6.7 Hz, 2H), 3.86 (s, 3H), 1.38 (t, J = 7.2 Hz, 3H); ^{13}C NMR (DMSO, 100 MHz): δ (ppm) 192.77, 164.80, 163.97, 156.52, 151.28, 145.19, 143.45, 130.37, 129.20, 128.20, 125.22, 121.61, 120.65, 111.28, 111.09, 109.19, 108.40, 52.75, 38.65, 14.44.

Methyl 10-(benzo[*d*]thiazol-2-yl)-7-ethyl-2-oxo-2,7-dihydropyrano[3,2-*c*]carbazole-3-carboxylate (**5**). Compound **4** (200 mg, 0.57 mmol), 2-aminothiophenol (108 mg, 0.86 mmol), and chlorotrimethylsilane (187 mg, 1.72 mmol) were combined in DMF, and the resulting mixture was refluxed for 6 hours. The mixture was cooled, and the solvent was evaporated. The product was extracted with DCM. The organic layer was washed with H_2O and brine, dried over Na_2SO_4, and concentrated to obtain the crude product. The product was purified by recrystallization from ethyl acetate. Yield: 77%; ^{1}H NMR ($CDCl_3$, 400 MHz): δ (ppm) 8.99 (d, J = 1.2 Hz, 1H), 8.66 (s, 1H), 8.30 (dd, J = 8.6, 1.7 Hz, 1H), 8.11 (d, J = 7.6 Hz, 1H), 7.95 (d, J = 7.4 Hz, 1H), 7.55 (d, J = 8.6 Hz, 1H), 7.48-7.53 (m, 2H), 7.37-7.43 (m, 1H), 7.31 (d, J = 8.6 Hz, 1H), 4.43 (q, J = 7.3 Hz, 2H), 3.99 (s, 3H), 1.52 (t, J = 7.2 Hz, 3H); ^{13}C NMR ($CDCl_3$, 100 MHz): δ (ppm) 168.38, 164.50, 156.76, 154.38, 152.73, 150.44, 144.61, 141.18, 135.22, 127.41, 127.35, 126.25, 126.09, 124.91, 123.29, 123.02, 121.63, 121.34, 112.64, 110.46, 110.11, 109.49, 106.74, 52.65, 38.58, 14.11.

10-(benzo[*d*]thiazol-2-yl)-7-ethyl-2-oxo-2,7-dihydropyrano[3,2-*c*]carbazole-3-carboxylic acid (**BPC1**). To a solution of **5** (100 mg, 0.22 mmol) in tetrahydrofuran (4 mL), an aqueous solution (1 mL) of lithium hydroxide (53 mg, 2.20 mmol) was added via a cannula. The reaction mixture was stirred at room temperature for 6 hours. The solvent was evaporated, and 5.0 mL of distilled water was added to the mixture. The aqueous portion was acidified with 37% hydrochloric acid at < 5°C until the pH was equal to 3. The resulting precipitate was collected by filtration, washed with distilled water followed by diethyl ether, and dried in vacuo. The product was obtained without further purification. Yield: 74%; ^{1}H NMR (DMSO, 400 MHz): δ (ppm) 13.00 (br s, 1H), 8.98 (s, 1H), 8.94 (s, 1H), 8.23 (dd, J = 8.6, 1.6 Hz, 1H), 8.16 (d, J = 8.0 Hz, 1H), 8.13 (d, J = 8.0 Hz, 1H), 7.98 (d, J = 8.6 Hz, 1H), 7.92 (d, J = 8.8 Hz, 1H), 7.76 (d, J = 8.6 Hz, 1H), 7.56 (t, J = 8.0 Hz, 1H), 7.46 (t, J = 7.6 Hz, 1H), 4.62 (q, J = 7.2 Hz, 2H), 1.40 (t, J = 7.2 Hz, 3H); ^{13}C NMR (DMSO, 100 MHz): δ (ppm) 168.23, 164.79, 161.62, 157.70, 154.29, 152.36, 151.00, 144.95, 141.67, 134.82, 129.77, 128.99, 127.12, 126.46, 125.70, 123.12, 122.79, 121.14, 111.64, 111.07, 109.08, 108.30, 38.60, 14.46.

N-(2-(2-(2-aminoethoxy)ethoxy)ethyl)-2-(1-(4-chlorobenzoyl)-5-methoxy-2-methyl-1*H*-indol-3-yl)acetamide (**8**). A mixture of **6** (200 mg, 0.56 mmol), **7** (91 mg, 0.62 mmol), and hydroxybenzotriazole (HOBt, 91 mg, 0.67 mmol) was dissolved in DMF (10 mL). To this mixture, *N,N*-diisopropylethylamine (145 mg, 1.12 mmol) was added, and the reaction mass was stirred under a nitrogen atmosphere for 10 minutes. After stirring, benzotriazol-1-yl-oxytripyrrolidinophosphonium hexafluorophosphate (349 mg, 0.67 mmol) was added, and the resulting mixture was stirred at room temperature for 12 hours under a nitrogen atmosphere. The solvent was evaporated, and the product was extracted with DCM. The organic layer was washed with H_2O and brine, dried over Na_2SO_4, and concentrated to obtain the crude product. The product was purified by flash column chromatography using DCM/MeOH (98:2) as the eluent. Yield: 37%; ^{1}H NMR ($CDCl_3$, 400 MHz): δ (ppm) 8.01 (s, 1H), 7.62 (d, J = 8.4 Hz, 2H), 7.46 (d, J = 8.6 Hz, 2H), 6.90 (d, J = 2.5 Hz, 1H) 6.82 (d, J = 9.1 Hz, 1H), 6.65 (dd, J = 8.9, 2.6 Hz, 1H) 6.20 (s, 1H), 3.79 (s, 3H), 3.62 (s, 2H), 3.60-3.64 (m, 8H), 3.30 (s, 4H), 2.36 (s, 3H); ^{13}C NMR ($CDCl_3$, 100 MHz): δ (ppm) 169.92, 168.27, 156.15, 139.54, 136.35, 133.52, 131.18, 130.86, 130.40, 129.20, 116.70, 115.02, 112.87, 111.97, 100.99, 70.07, 69.73, 55.71, 39.22, 32.18, 13.28.

10-(benzo[*d*]thiazol-2-yl)-*N*-(2-(2-(2-(2-(1-(4-chlorobenzoyl)-5-methoxy-2-methyl-1*H*-indol-3-yl)acetamido)ethoxy)ethoxy)ethyl)-7-ethyl-2-oxo-2,7-dihydropyrano[3,2-*c*]carbazole-3-carboxamide (**IBPC1**). A mixture of **BPC1** (42 mg, 0.095 mmol), *N,N′*-dicyclohexylcarbodiimide (39 mg, 0.19 mmol), and HOBt (25 mg, 0.19 mmol) in DMF (mL) was stirred for 2 hours at room temperature. To this mixture, **6** (51 mg, 0.11 mmol) was added and stirred overnight. The solvent was evaporated, and the reaction mixture was dissolved in acetonitrile; then, byproduct urea was removed by filtration. The filtrate was concentrated under reduced pressure. The product was purified by flash column chromatography using DCM/MeOH (98:2) as the eluent. Yield: 24%; ^{1}H NMR ($CDCl_3$, 400 MHz): δ (ppm) 9.21 (s, 1H), 8.78 (s, 1H), 8.42 (dd, J = 8.6, 1.8 Hz, 1H), 8.12 (d, J = 8.2 Hz, 1H), 7.95 (d, J = 7.8 Hz, 1H), 7.66 (d, J = 8.6 Hz, 1H), 7.60 (d, J = 8.6 Hz, 1H), 7.49-7.55 (m, 2H), 7.37-7.43 (m, 3H), 7.16-7.22 (m, 2H), 6.71-6.81 (m, 3H), 5.35 (d, J = 5.6 Hz, 2H), 4.49 (q, J = 7.4 Hz, 2H), 4.00 (s, 3H), 3.71-3.75 (m, 3H), 3.63 (s, 2H), 3.57-3.60 (m, 3H), 3.44-3.51 (m, 4H), 3.42 (s, 2H), 2.35 (t, J = 7.4 Hz, 3H), 1.54 (t, J = 7.2 Hz, 3H); ^{13}C NMR ($CDCl_3$, 100 MHz): δ (ppm) 170.09, 168.38, 167.43, 165.32, 156.76, 156.46, 155.15, 150.74, 144.61, 141.46, 140.24, 136.62, 134.54, 133.25, 130.92, 130.21, 130.14, 129.85, 127.16, 127.99, 126.50, 126.09, 124.66, 123.64, 123.54, 121.46, 121.39, 115.25, 112.87, 112.64, 111.86, 110.68, 110.04, 109.59, 107.27, 101.19, 70.14, 69.87, 55.71, 52.05, 39.18, 38.62, 32.21, 14.10, 13.27; HRMS (ESI$^+$): m/z calculated for $C_{50}H_{44}O_8N_5ClS$: 909.2594, found: 909.2055.

Water solubility. A small amount of **IBPC1** was dissolved in DMSO to prepare the stock solutions (1.0×10^{-2} M). The solution was diluted to between 8.0×10^{-6} and 2.0×10^{-7} M and added to a cuvette containing 2.0 mL of PBS (phosphate-buffered saline) buffer (10 mM, pH 7.4) using a microsyringe. In all cases, the concentration of DMSO in the buffer was maintained at 0.1%. The plots of fluorescence intensity against the dye concentration were linear at low concentrations and showed downward curvature at higher concentrations (Figure S1). The maximum concentration in the linear region was interpreted as the solubility. The solubility of **IBPC1** in PBS buffer was approximately 2.0 μM.

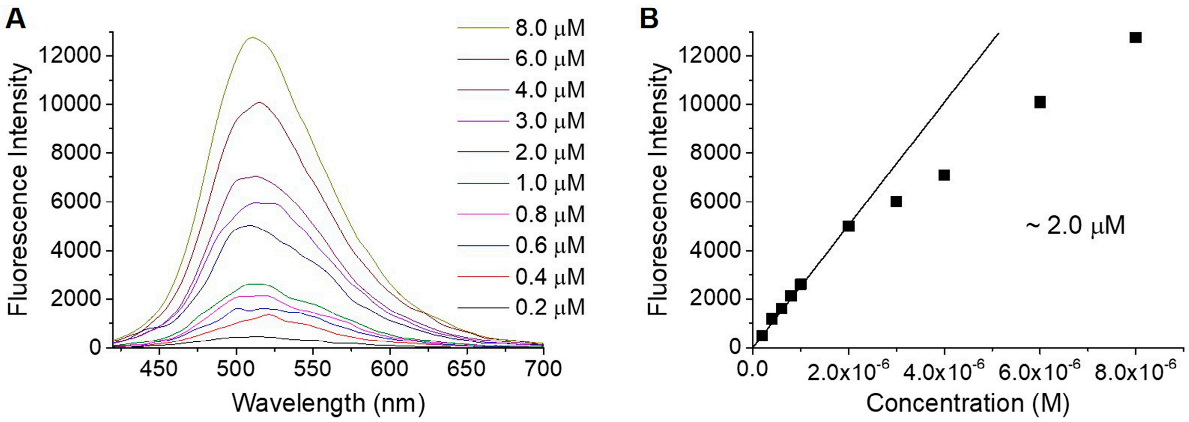

Figure S1. (A) Changes in fluorescence spectrum according to the concentration of **IBPC1**. (B) Plot of fluorescence intensity against concentration for **IBPC1** in phosphate buffer. The excitation wavelength was 380 nm.

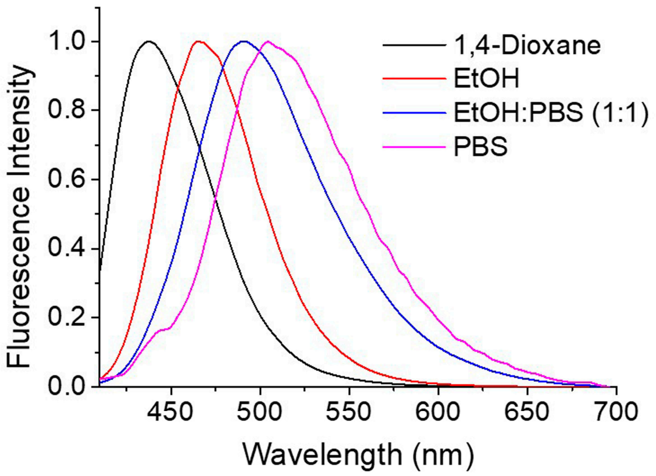

Figure S2. Normalized emission spectra of **IBPC1** in 1,4-dioxane, EtOH, EtOH: PBS (v/v, 1:1) and PBS buffer

Table S1. Photophysical properties of **IBPC1** in various solvents.

Solvent	(1)λ_{max}^{abs}	(2)λ_{max}^{fl}	(3)Φ	(4)ε
1,4-Dioxane	375	438	0.43	1.33
EtOH	380	465	0.31	1.87
EtOH:PBS (1:1)	388	491	0.20	1.70
PBS	378	505	0.02	0.70

λ_{max} of the (1) absorption and (2) emission spectra in nm. (3) Fluorescence quantum yield. The uncertainty was ± 15%. (4) Molar extinction coefficient in 10^{-4} $M^{-1}cm^{-1}$.

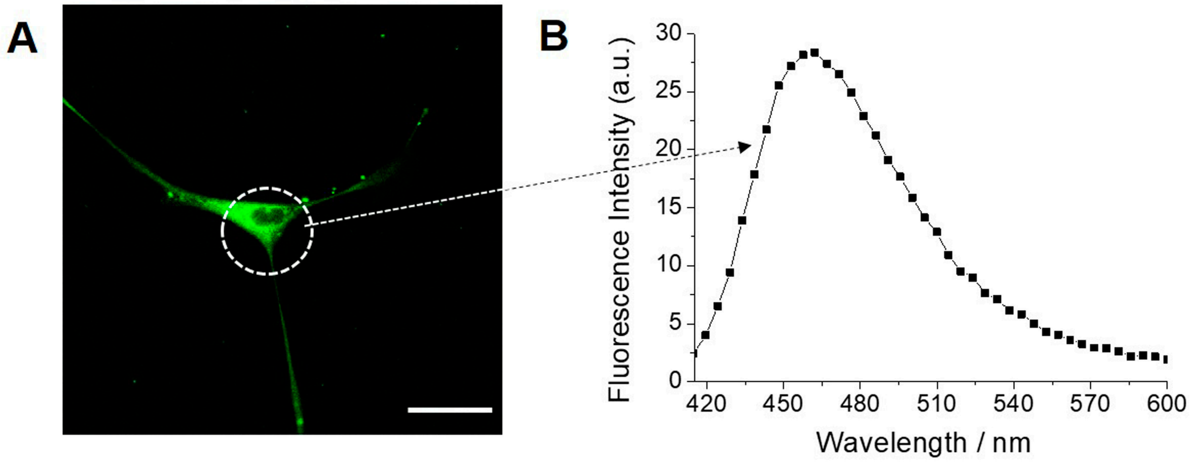

Figure S3. (A) Fluorescence image of 2 μM **IBPC1** in nucleus pulposus cells (NPCs). Scale bar: 60 μm. (B) Fluorescence spectra of **IBPC1** in NP cells. The excitation wavelengths were 405 nm.

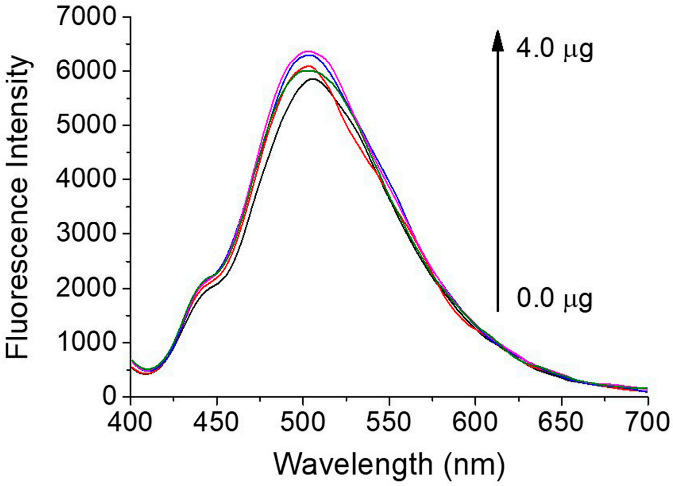

Figure S4. Changes in fluorescence spectrum of **IBPC1** according to the concentration of cyclooxygenase-1 (COX-1) (0-4 μg/mL).

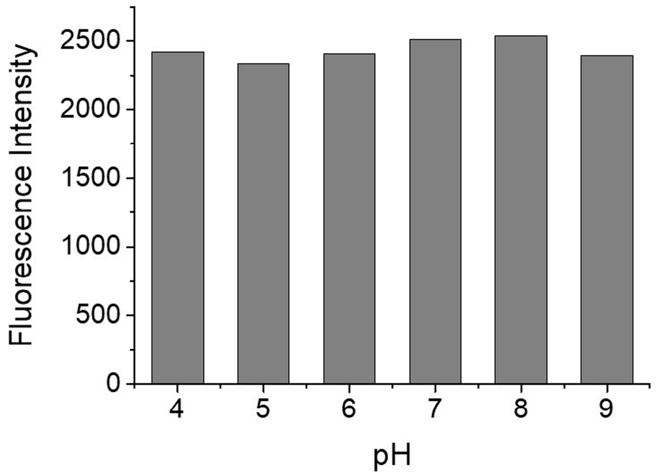

Figure S5. Effect of pH (4.0-9.0) on the fluorescence intensity of 2 μM **IBPC1** in universal buffer (0.1 M citric acid, 0.1 M KH_2PO_4, 0.1 M $Na_2B_4O_7$, 0.1 M Tris, 0.1 M KCl). The excitation wavelength was 380 nm.

Cell viability. To confirm that the **IBPC1** couldn't affect the viability of nucleus pulposus cells (NPCs), we used CCK-8 kit (Cell Counting Kit-8, Dojindo, Japan) according to the manufacture's protocol.

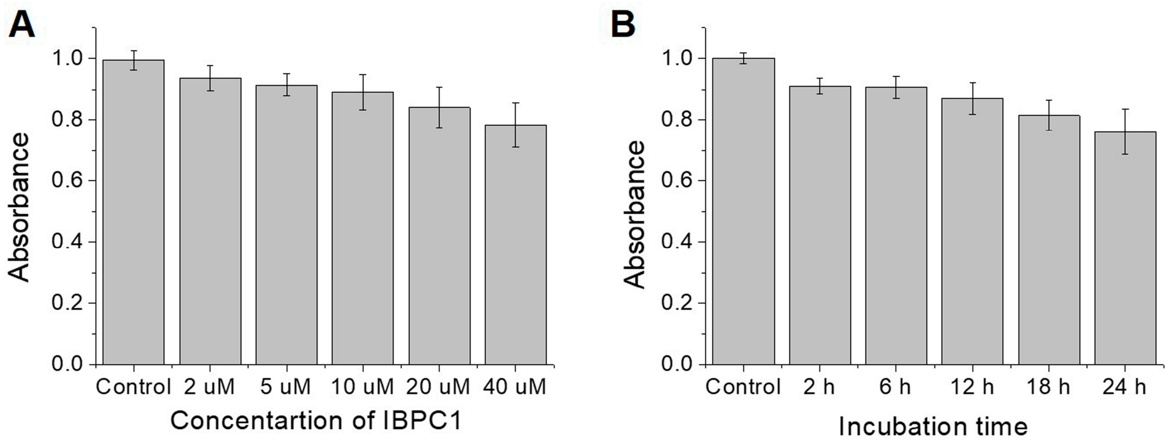

Figure S6. Viability of nucleus pulposus cells (NPCs) in the presence of **IBPC1** as measured using a Cell Counting Kit-8 assay kit. The cells were incubated with (A) 0-40 μM **IBPC1** for 10 h and (B) 10 μM **IBPC1** for 2, 6, 12, 18, and 24 h. Five independent experiments were performed.

Photostability. Photostability of **IBPC1** in nucleus pulposus cells (NPCs) was determined by monitoring the changes in fluorescence intensity with time at three designated positions. The digitized intensity was recorded with 2-second intervals for 2000 seconds using xyt mode. The fluorescence intensities were collected at 400-600 nm upon excitation at 405 nm.

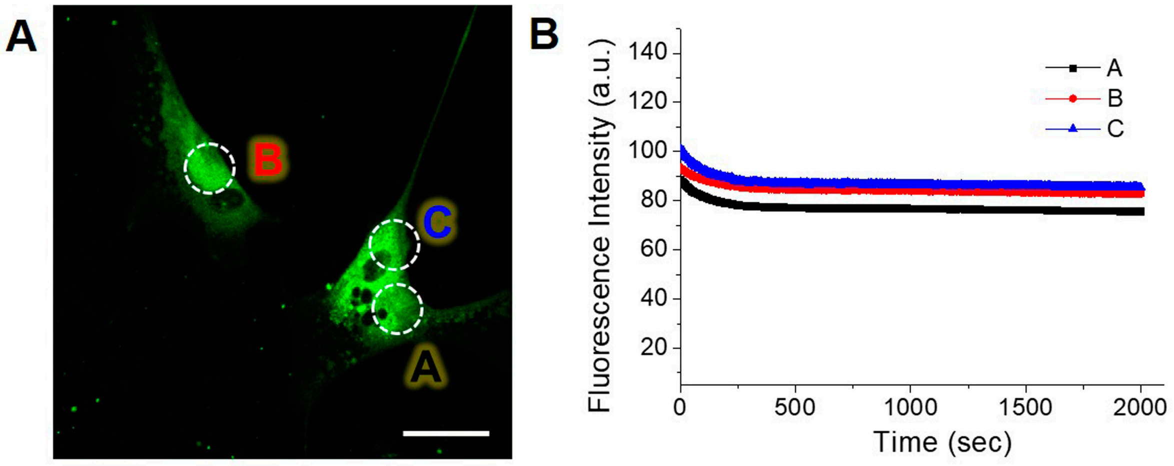

Figure S7. (A) Confocal fluorescence images of **IBPC1**-labeled (2 μM) nucleus pulposus cells (NPCs). (B) The relative fluorescence intensity from A-C in (A) as a function of time. Cells shown are representative images from replicate experiments (n = 5).

Co-localization experiment. For the co-localization experiments was conducted by co-staining the nucleus pulposus cells (NPCs) with **IBPC1** (5.0 μM) for 30 min. **IBPC1**-labeled NPCs were washed with DPBS, and the cells were fixed with 4 % paraformaldehyde in DPBS for 15 min. Then, cells were permeabilized with 0.2% Triton X-100 for 5 min and washed three times with DPBS. The cells were blocked with 2% (w/v) bovine serum albumin (BSA) in DPBS for 1 h at room temperature with shaking, and then incubated with primary antibody to COX-2 (1:400 in DPBS containing 2 % BSA) for overnight at 4 °C with shaking. After washing three times with TBST buffer (20 mM Tris-HCl, 150 mM NaCl, 0.1 % tween 20, pH 7.5), secondary fluorescent antibodies (Alexa 594) were incubated for 2 h at room temperature with shaking. Then, cells were washed three times with TBST buffer for fluorescence imaging. Images were obtained by collecting the emissions at 400-480 (OBPC1, λ_{ex} = 405 nm) and 620-700 nm (COX-2, λ_{ex} = 594 nm), respectively. The background images were corrected, and the distribution of pixels in the images acquired in the green and red channels, respectively, was compared by using scatter gram. The Pearson's colocalization coefficient (*A*) was calculated using LAS AF software.

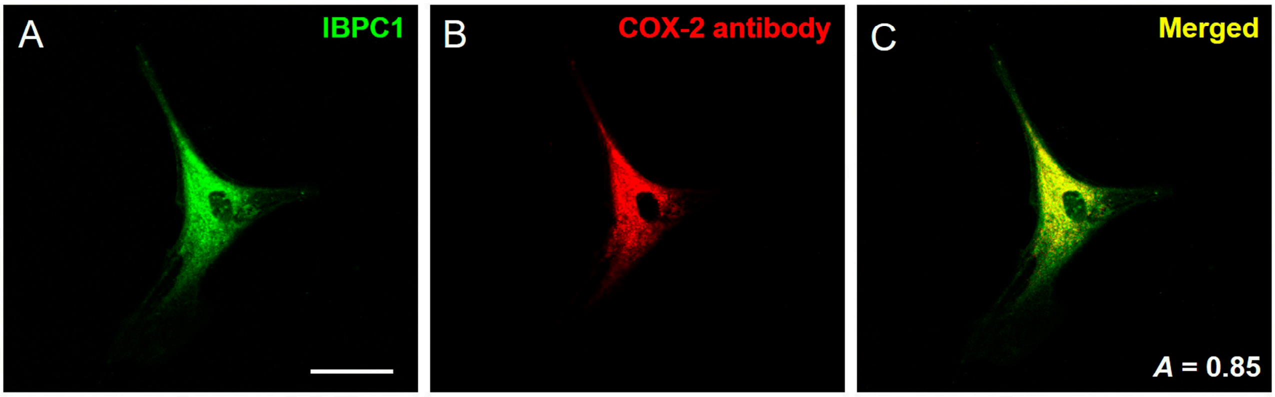

Figure S8. Co-localization fluorescence images of nucleus pulposus cells (NPCs) incubated with (A) **IBPC1** (5 μM) for 30 min and then with (B) COX-2 antibody for 1 day at 4 ºC. (C) The merged image and the Pearson correlation coefficient calculated accordingly. Scale bars: 60 μm.

RNA isolation and quantitative real-time PCR (qPCR). Nucleus pulposus cells (NPCs) used for quantitative real-time PCR (qPCR) were grown in tissue culture dish Ø 60 mm (#93060, TPP). RNA extraction was performed in each 60 mm cell culture dish using 500 μL Trizol reagent (15596018, Thermo Fisher). RNA concentration was measured by Nanodrop, and complementary DNA synthesis (cDNA) for qPCR was performed with a conventional PCR thermocycler (T100, BioRad) by reverse transcription premix (#25081, iNtRON Biotechnology). cDNA synthesis conditions are 45°C for 60 min and RTase inactivation step at 95°C for 5 min. qPCR was diluted according to Nuclease Free Water (AM9937, Thermo Fisher) to match the RNA concentration of 1 μg, and was used for quantification. Primers were used as follows. As a control target, 18s primer (F: 5'-GTAACCCGTT-GAACCCCATT- 3' , R: 5'-CCATCCAATCGGTAGTAGCG- 3'), Cox-2 primer (F: 5'-CGGTGAAACTCTGGCTAGACAG- 3', R: 5'- GCAAACCGTAGATGCTCAGGGA- 3'), Cox-1 primer (F: 5'-GATGAGCAGCTTTTCCAGACGAC-3', R: 5'-AACTGGACACCGAACAGCAGCT-3'). A total of 5 groups were control, LPS (50 ng/mL) + IFN-γ, LPS (250 ng/mL) + IFN-γ, LPS (500 ng/mL) + IFN-γ, and LPS (500 ng/mL) + IFN-γ + COX-2 inhibitor cDNA for each group was used. When samples and primers are prepared in a microplate by qPCR SYBER Green (#4368708, Thermo Fisher) protocol, proceed under the following conditions using Applied Biosystems StepOne Real-Time PCR System (#4376600, Thermo Fisher). Follow as a condition: denaturation at 95 °C for 10 min, followed by 40 cycles at 95 °C for 15 s. Data were analyzed according to the ΔΔCt method, and primers were synthesized from Bioneer.

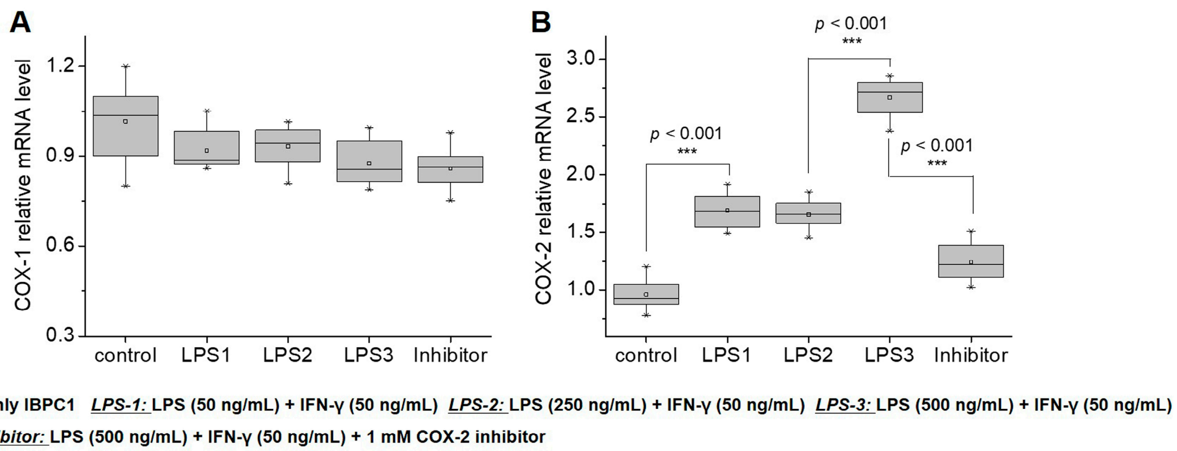

Figure S9. qPCR analysis of nucleus pulposus cells (NPCs) that were pretreated with LPS. Relative

mRNA expression levels of (A) COX-1 and (B) COX-2 were normalized against the corresponding levels of 18s rRNA. Asterisks indicate statistical significance (***p < 0.001). The cells shown are representative images from replicate experiments (n = 5).

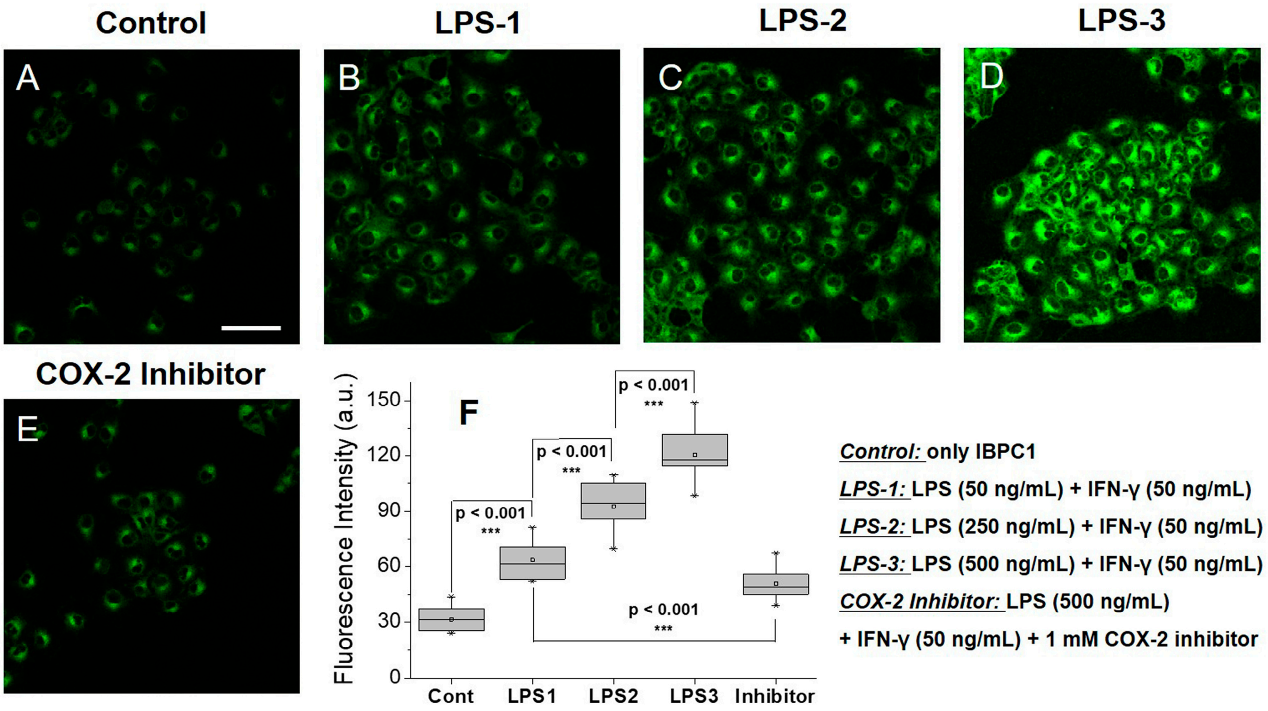

Figure S10. (A-E) Fluorescence images of **IBPC1** by interaction with COX-2 in HeLa cells. Cells were labeled with 2 μM **IBPC1** (A) and pretreated with (B) 50, (C) 250, (D) 500 ng/mL LPS, 50 ng/mL IFN-γ for 5 hr. (E) 1 mM COX-2 inhibitor was treated for 1 hr after being pretreated with 500 ng/mL LPS, 50 ng/mL IFN-γ for 5 hr. (F) Box-plot of fluorescent intensity in A-E. The fluorescent intensities were collected at 400–600 nm upon excitation at 405 nm and measured in 500 randomly chosen regions. Asterisks indicate statistical significance (***p < 0.001). Cells shown are representative images from replicate experiments (n =5). Scale bars: (a-e) 60 μm.

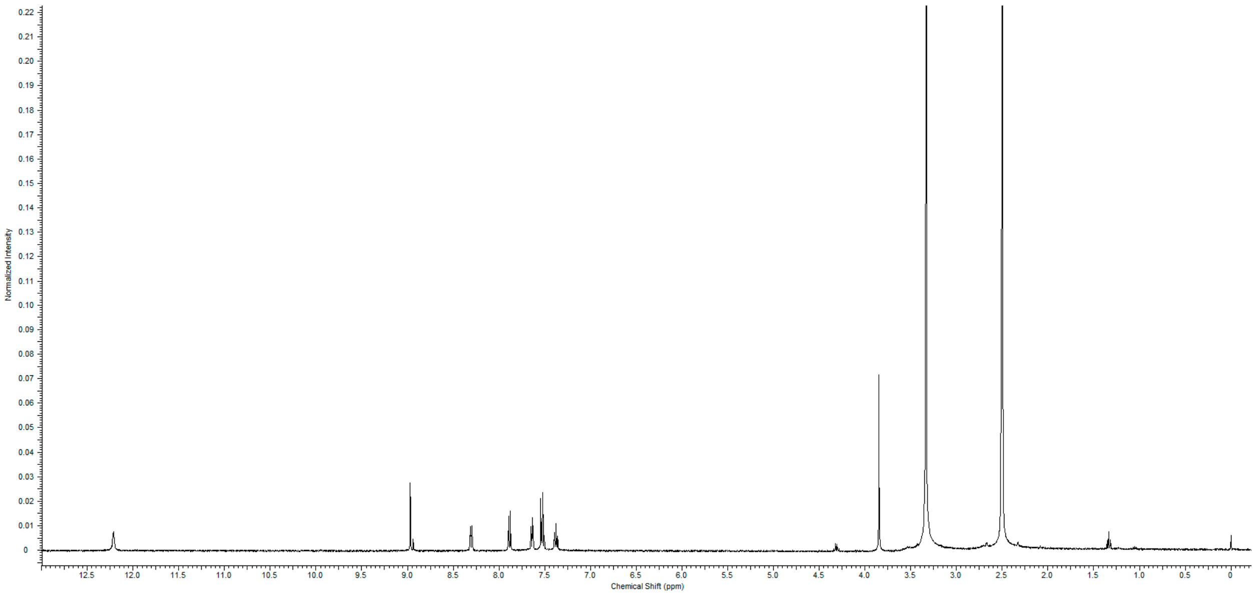

Figure S11. ^{1}H-NMR spectrum (400 MHz) of **2** in DMSO.

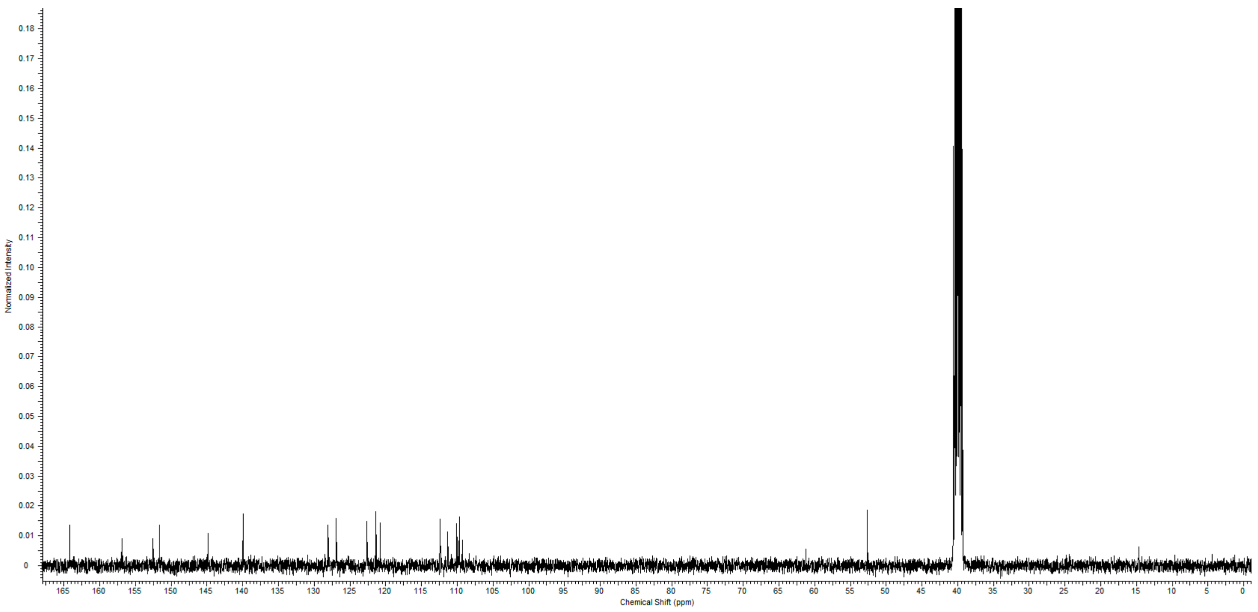

Figure S12. ^{13}C-NMR spectrum (400 MHz) of **2** in DMSO.

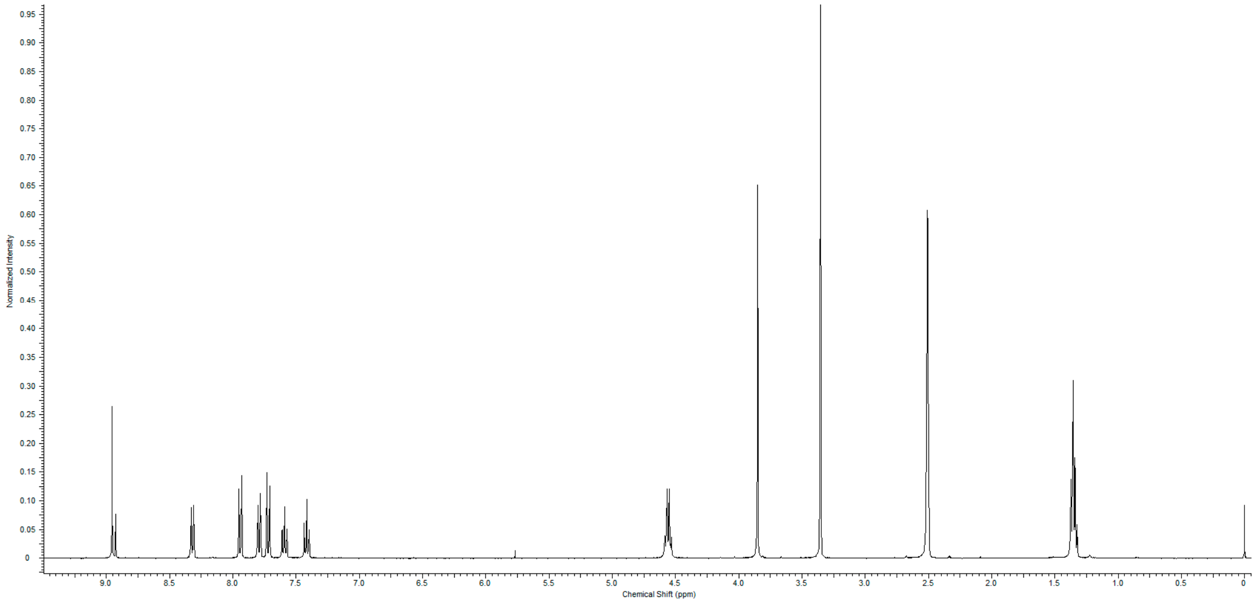

Figure S13. ^{1}H-NMR spectrum (400 MHz) of **3** in DMSO.

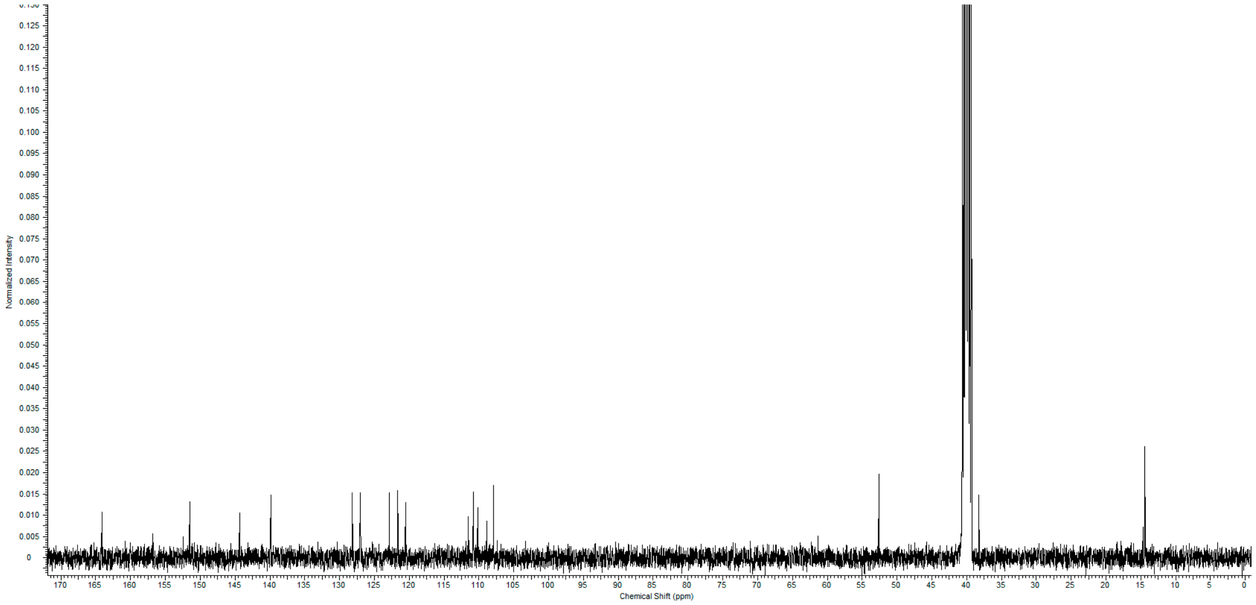

Figure S14. ^{13}C-NMR spectrum (400 MHz) of **3** in DMSO.

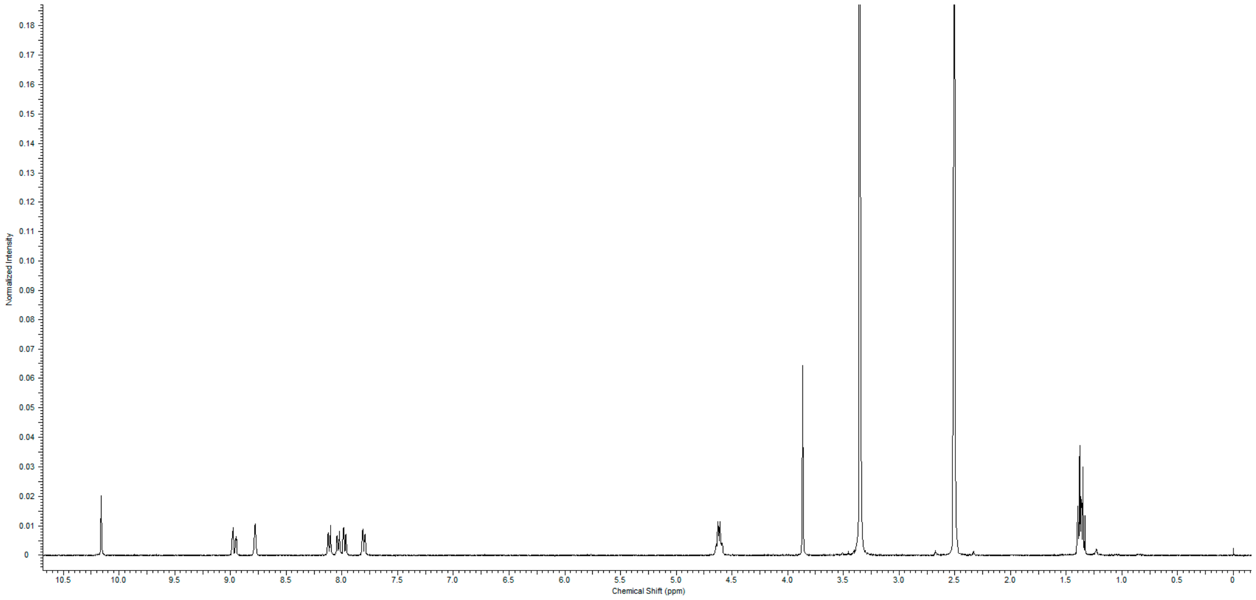

Figure S15. ^{1}H-NMR spectrum (400 MHz) of **4** in DMSO.

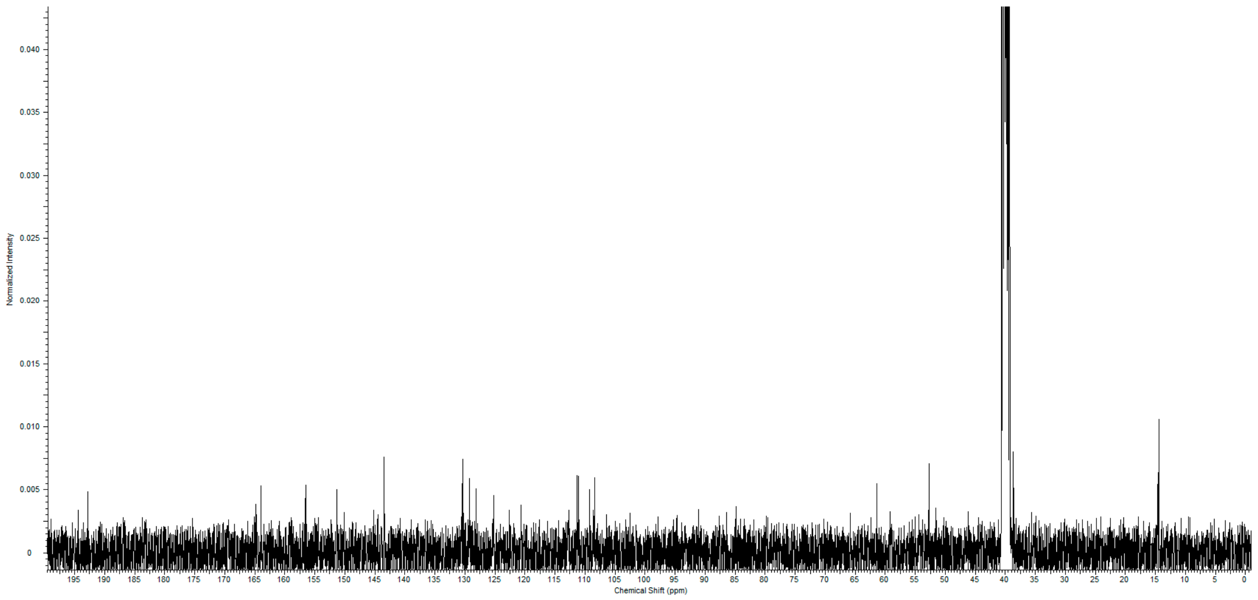

Figure S16. ^{13}C-NMR spectrum (100 MHz) of **4** in DMSO.

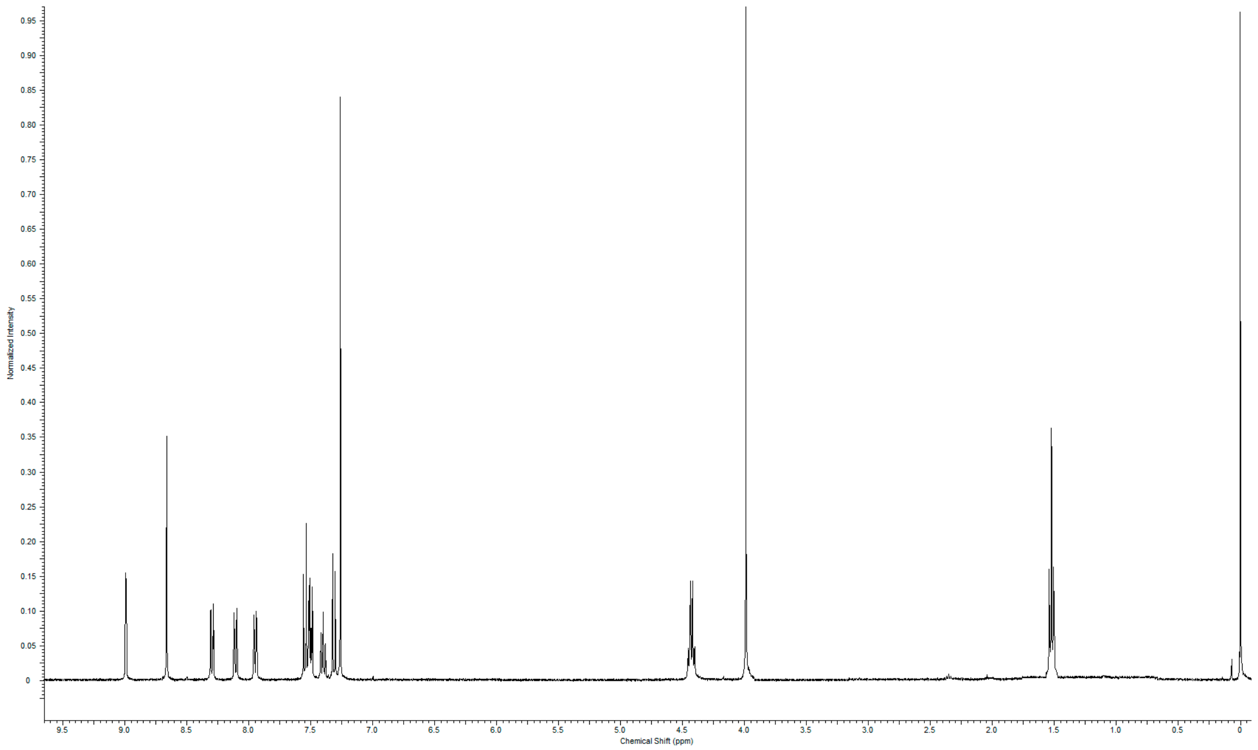

Figure S17. ^{1}H-NMR spectrum (400 MHz) of **5** in $CDCl_3$.

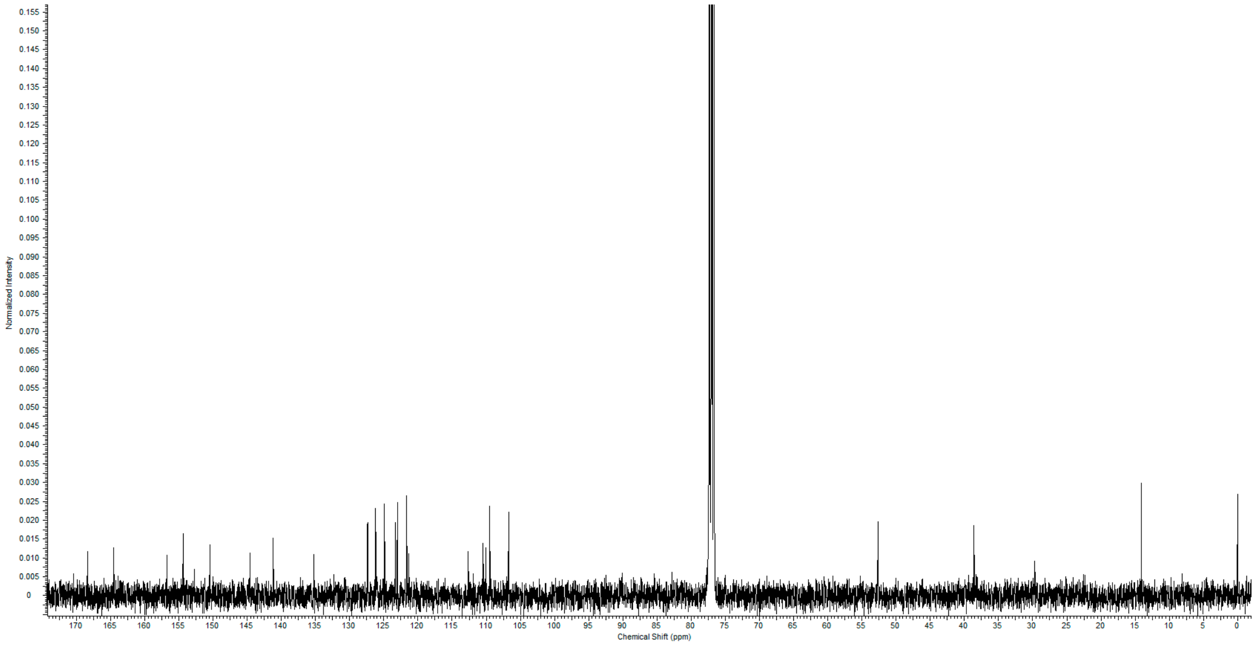

Figure S18. ^{13}C-NMR spectrum (100 MHz) of **5** in $CDCl_3$.

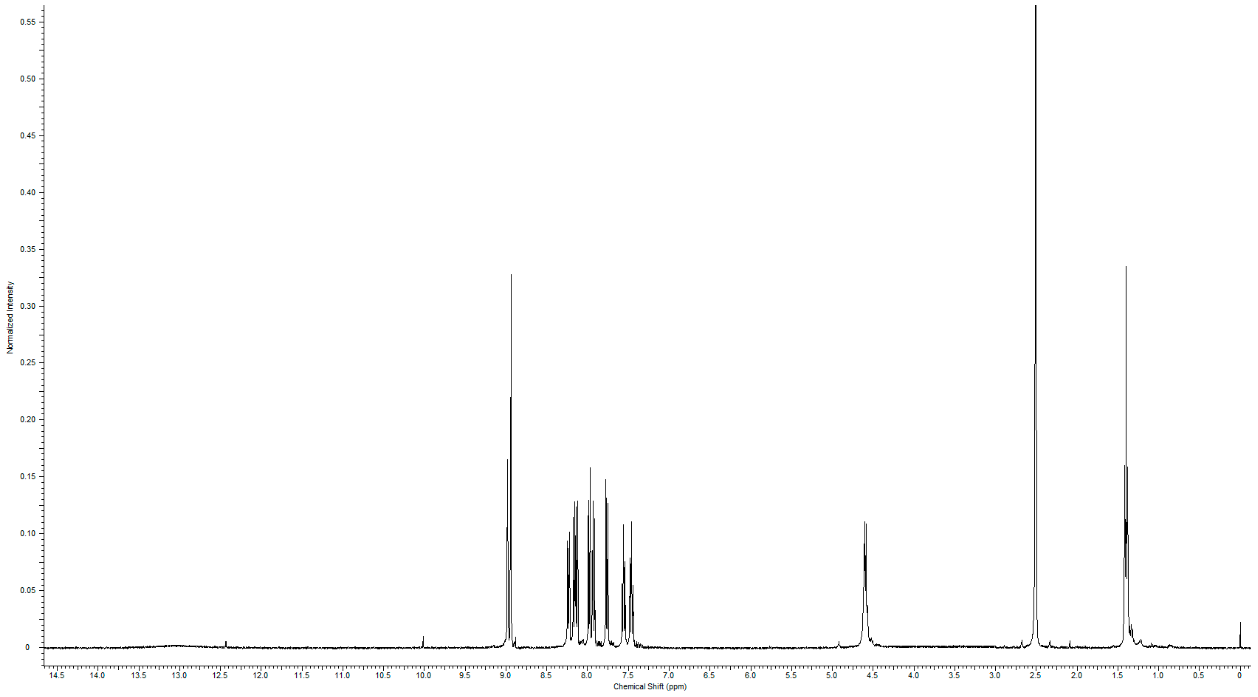

Figure S19. ^{1}H-NMR spectrum (400 MHz) of **BPC1** in DMSO.

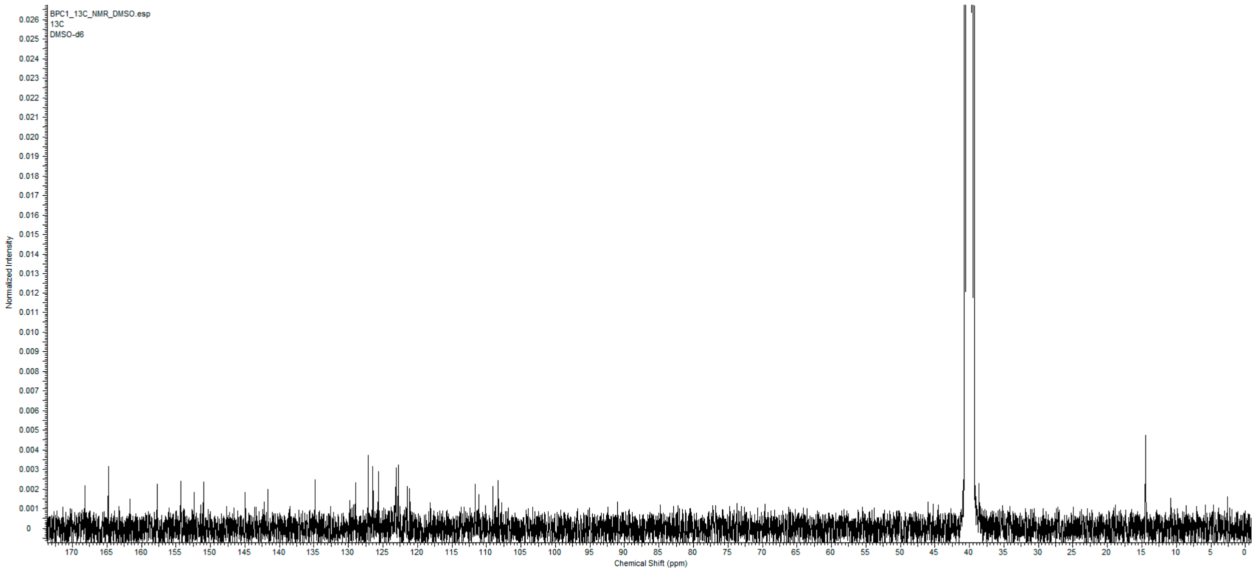

Figure S20. ^{13}C-NMR spectrum (100 MHz) of **BPC1** in DMSO.

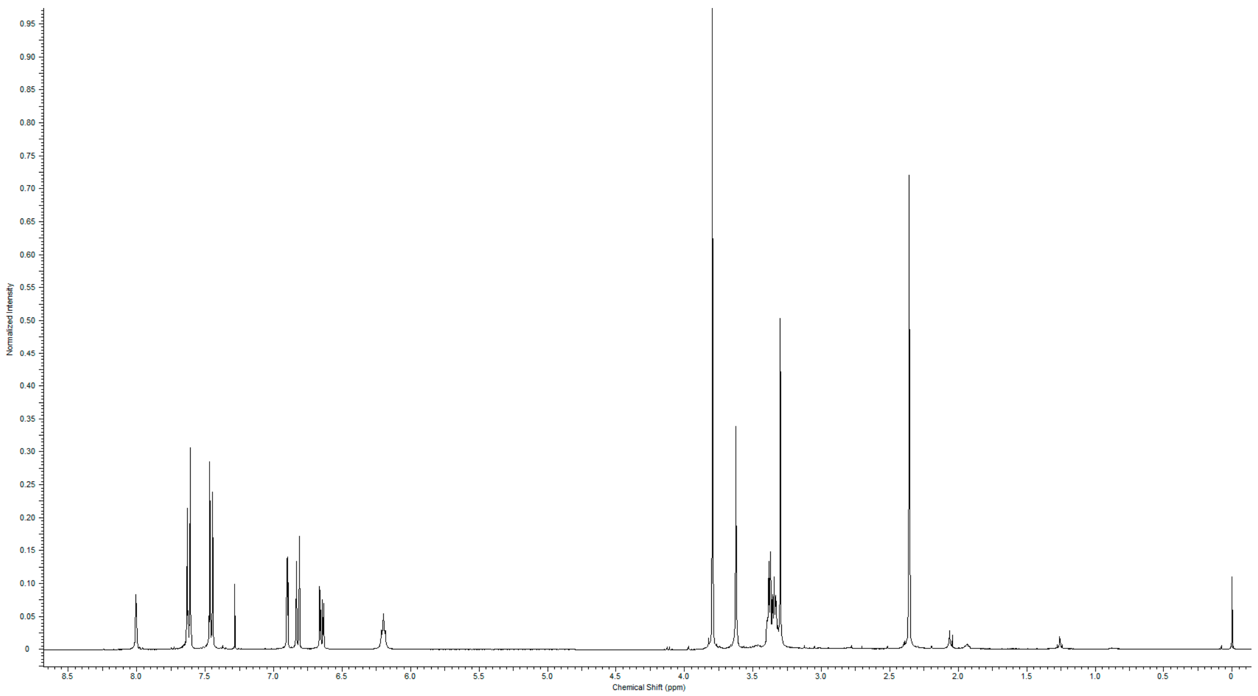

Figure S21. ^{1}H-NMR spectrum (400 MHz) of **8** in $CDCl_3$.

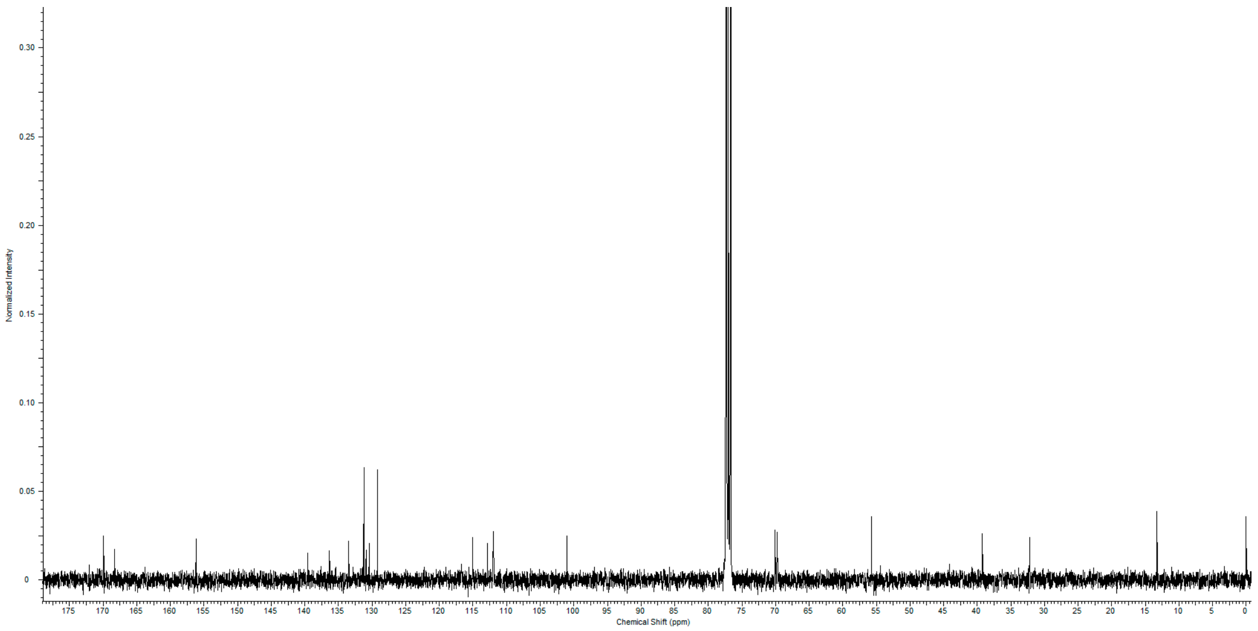

Figure S22. ^{13}C-NMR spectrum (400 MHz) of **8** in $CDCl_3$.

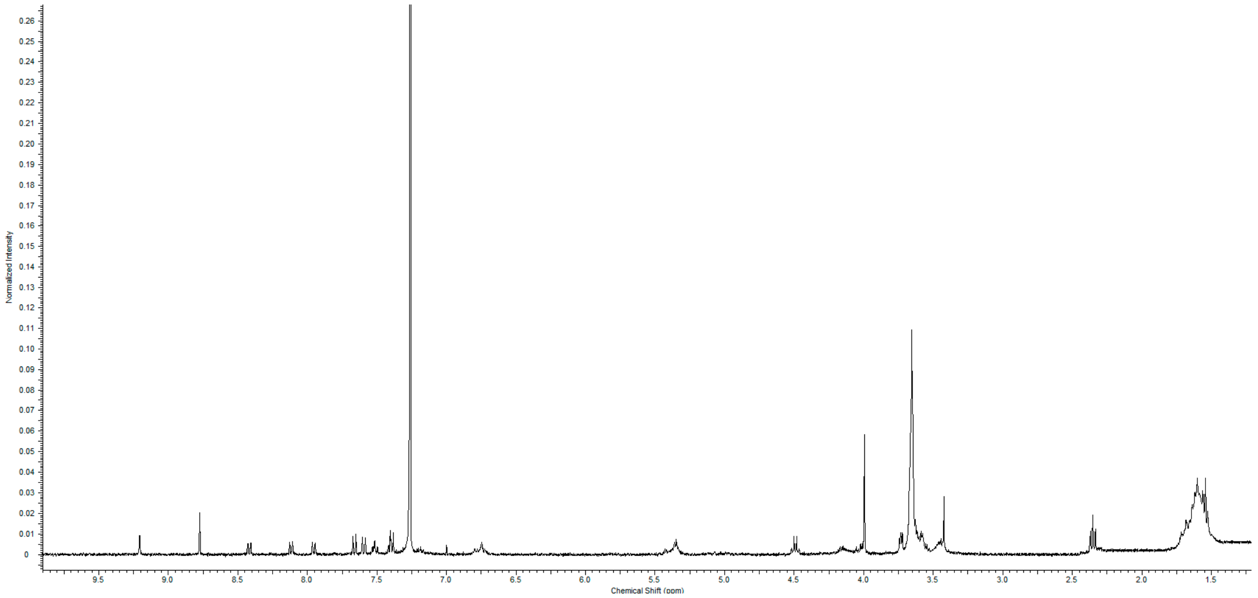

Figure S23. ^{1}H-NMR spectrum (400 MHz) of **IBPC1** in $CDCl_3$.

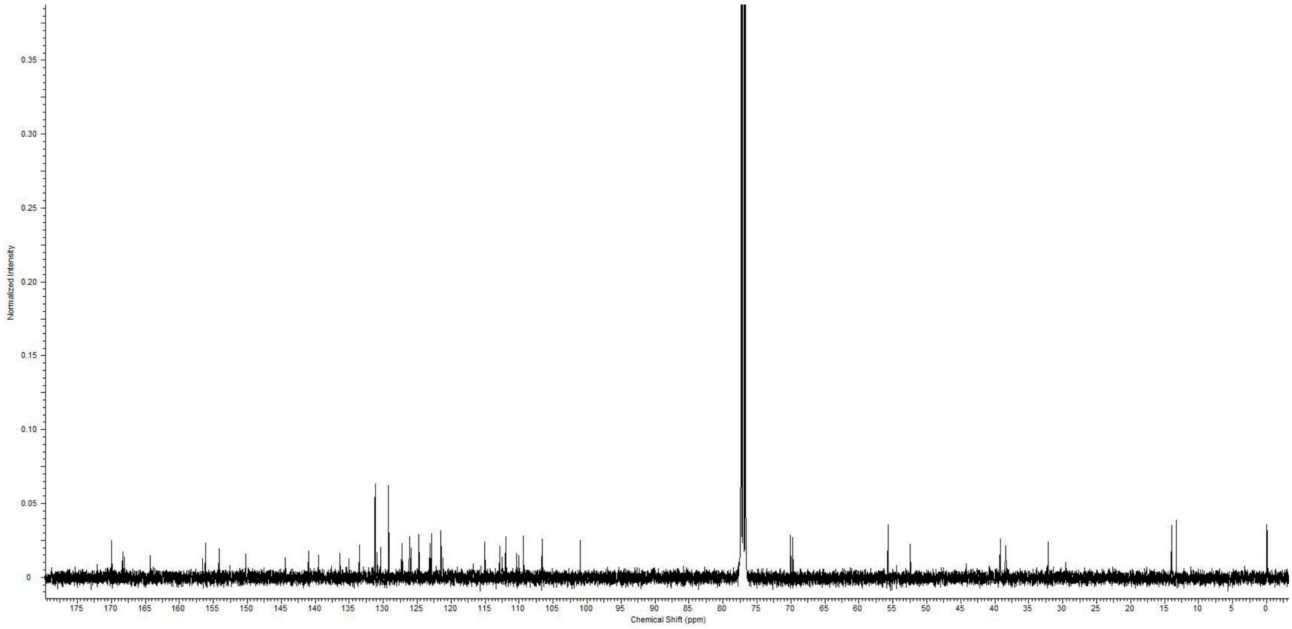

Figure S24. ^{13}C-NMR spectrum (100 MHz) of **IBPC1** in $CDCl_3$.

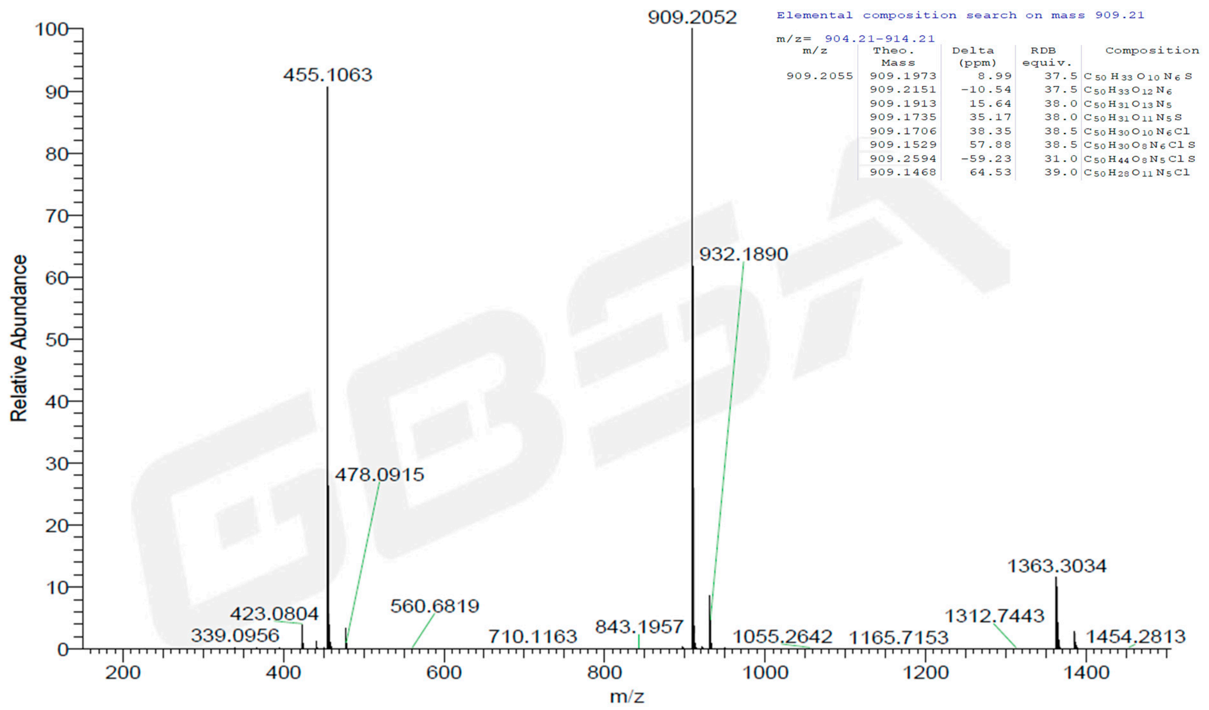

Figure S25. HRMS spectrum of **IBPC1**.

Reference

[1] S. M. Bhosale, A. A. Momin and R. S. Kusurkar, *Tetrahedron* **2012**, *68* (32), 6420-6426.
